# Assessments of Alpha-Amylase Inhibitory Potential of Tagetes Flavonoids through In Vitro, Molecular Docking, and Molecular Dynamics Simulation Studies

**DOI:** 10.3390/ijms241210195

**Published:** 2023-06-15

**Authors:** Gamal A. Mohamed, Abdelsattar M. Omar, Moustafa E. El-Araby, Shaza Mass, Sabrin R. M. Ibrahim

**Affiliations:** 1 Department of Natural Products and Alternative Medicine, Faculty of Pharmacy, King Abdulaziz University, Jeddah 21589, Saudi Arabia; 2 Department of Pharmaceutical Chemistry, Faculty of Pharmacy, King Abdulaziz University, Jeddah 21589, Saudi Arabia; asmansour@kau.edu.sa (A.M.O.); madaoud@kau.edu.sa (M.E.E.-A.); shazaamass@gmail.com (S.M.); 3 Center for Artificial Intelligence in Precision Medicines, King Abdulaziz University, Jeddah 21589, Saudi Arabia; 4 Department of Chemistry, Preparatory Year Program, Batterjee Medical College, Jeddah 21442, Saudi Arabia; sabrin.ibrahim@bmc.edu.sa; 5 Department of Pharmacognosy, Faculty of Pharmacy, Assiut University, Assiut 71526, Egypt

**Keywords:** *Tagetes minuta*, alpha-amylase inhibitors, diabetes, molecular docking, life on land, molecular dynamics, drug discovery, health and wellbeing

## Abstract

Diabetes is a chronic fast-growing metabolic disorder that is characterized by high blood glucose levels. *Tagetes minuta* L. has been used as a traditional remedy for various illnesses for many years, and, furthermore, its oil is used in the perfume and flavor industries. *T. minuta* contains various metabolites, such as flavonoids, thiophenes, terpenes, sterols, and phenolics, with varied bioactivities. Flavonoids can inhibit carbohydrate-digesting enzymes, such as alpha-amylase, which is a convenient dietary strategy for controlling hyperglycemia. In the current investigation, the isolated flavonoids quercetagetin-6-O-(6-O-caffeoyl-β-D-glucopyranoside), quercetagetin-7-O-β-D-glucopyranoside, quercetagetin-6-O-β-D-glucopyranoside, minutaside A, patuletin-7-O-β-D-glucopyranoside, quercetagetin-7-methoxy-6-O-β-D-glucopyranoside, tagenols A and B, quercetagetin-3,7-dimethoxy-6-O-β-D-glucopyranoside, patuletin, quercetin-3,6-dimethyl ether, and quercetin-3-methyl ether from *T. minuta* were assessed for their alpha-amylase inhibition (AAI) efficacy using an in vitro assay, as well as molecular docking, dynamics simulation, and ADMET analyses. Our findings show that quercetagetin-6-O-(6-O-caffeoyl-β-D-glucopyranoside) (**1**), quercetagetin-7-O-β-D-glucopyranoside (**2**), quercetagetin-6-O-β-D-glucopyranoside (**3**), minutaside A (**4**), patuletin-7-O-β-D-glucopyranoside (**5**), and quercetagetin-7-methoxy-6-O-β-D-glucopyranoside (**6**) had a notable AAI capacity (IC_50_s ranged from 7.8 to 10.1 μM) compared to acarbose (IC_50_ 7.1 μM). Furthermore, these compounds with the highest binding affinity among the tested flavonoids revealed high docking scores for AA (ranging from −12.171 to 13.882 kcal/mol) compared to that of acarbose (−14.668 kcal/mol). In MDS, these compounds were observed to show maximum stability and the greatest binding free energy, suggesting that they may contend with native ligands. In addition, the ADMET analysis showed that these active compounds had a broad span of drug-like, pharmacokinetic, and physicochemical features and did not possess any considerable undesired effects. The current results suggest the potential of these metabolites as AAI candidates. However, further in vivo and mechanistic studies are warranted to specify the efficacy of these metabolites.

## 1. Introduction

Inadequate insulin production, hyperglycemia, and decreased insulin utilization all contribute to DM (diabetes mellitus), which is a highly prevalent chronic metabolic condition. Usually, it is accompanied by disrupted protein, lipid, and carbohydrate metabolism [1]. These metabolic disorders lead to severe injuries to the nerves, heart, eyes, blood vessels, and kidneys [2]. DM includes type I and II DM. In type I DM, the β-cell destruction results in a drop in the amount of circulating insulin. However, most individuals with diabetes have type II diabetes, which is typically characterized by insulin resistance in peripheral tissues and a reduction in insulin responsiveness [1,2,3]. In addition to the reduced cellular uptake of glucose from blood due to insulin deficiency, oxidative stress is one of the major causes of type II DM [3]. Endogenous antioxidants are the primary line of protection in the human body, and any disruption of this line of defense results in OS through the release of free radicals and reactive oxygen species (ROS). Increased ROS levels are crucial to the emergence and advancement of type II DM [4].

Diabetes is a major global health concern with high morbidity and mortality risks, affecting millions of people worldwide [5]. In 2014, 422 million people had diabetes, compared to 108 million in 1980, and it is predicted to increase to 640 million by 2040, with 1.5 million deaths annually, mostly in middle- and low-income countries [2,5].

Therefore, consuming antioxidants and regulating carbohydrate intake by inhibiting carbohydrate-metabolizing enzymes, such as alpha-amylase, are regarded as beneficial approaches in controlling hyperglycemia, especially in type II DM [6,7]. Synthetic antioxidative agents (e.g., BHA (butylated hydroxyanisole) and BHT (butylated hydroxytoluene)) and commercially available alpha-amylase inhibitors (AAIs), such as miglitol, voglibose, and acarbose, can have adverse side effects, including heart failure, flatulence, and diarrhea [8,9]. Consequently, a vigorous effort has been undertaken to identify natural metabolites with potential therapeutic efficacy to counteract the drawbacks of diabetes. Different traditional medicines rely on the usage of medicinal plants and their phyto-constituents/formulations for treating diabetes; however, there are no scientific data to support these folkloric claims [10]. Many natural products, such as phenolic compounds, flavonoids, alkaloids, terpenoids, coumarins, and glycosides, are known to possess antidiabetic potential through diverse mechanisms, such as alpha-amylase and alpha-glucosidase inhibition, glucose uptake modulation, stimulating insulin secretion and beta-cell proliferation, and controlling insulin resistance and oxidative stress regulation [11,12]. Many plant extracts are rich in flavonoids/phenolics with powerful antioxidant and antidiabetic properties [13] that could have promising advantages and a potential contribution as a prophylactic and/or in the treatment of diabetes and its implications. 

The genus *Tagetes* L. (marigolds, Asteraceae) comprises 56 species of cultivated or wild, perennial, and annual herbaceous plants [14]. They are typically found in Asia, America, Argentina, Africa, Australia, and Europe [15,16]. *T. patula*, *T. minuta*, *T. tenuifolia*, and *T. erecta* are the most extensively studied species that have diverse bioactivities and agricultural applications [15]. In Mexico, marigolds are used as antiseptics, analgesics, diuretics, carminatives, stimulants, vermin repellents, and worm expellant. They are also utilized for insect repelling and food seasoning in America, as offerings to gods during Indian ceremonies, and in death ceremonies in Guatemala and Mexico [17]. These species provide a valuable commercial source of essential oils and metabolites with unique bioactivities for the food, agrochemical, nutritional supplement, colorant, and cosmetic industries [17]. 

*Tagetes minuta* L. has been traditionally used as a remedy for various illnesses, including calluses, coughs, dislodging congestion, chest infections, catarrh, bunions, cuts, colds, and wounds [18]. The infusion of its leaves is also known to be useful for stomach and intestinal disorders [19]. The plant oil of *T. minuta* has gained considerable industrial interest due to its unique aroma, commonly used in the perfume and flavor industries [16]. Studies have identified various metabolites in *T. minuta*, such as flavonoids, thiophenes, terpenes, sterols, fatty acids, and phenolics, with diverse bioactivities [20,21]. Reported flavonoids from this plant include flavonol O-glycosides and flavonols [6,17].

Additionally, some *T. minuta* flavonoids have been reported to have different bio-activities. For example, quercetagetin-7-O-β-D-glucopyranoside (IC_50_ 3.92 μg/mL for DPPH and %inhibition 82.85% for superoxide anion), patuletin-7-O-β-D-glucopyranoside (IC_50_ 12.1 μg/mL for DPPH), and quercetagetin-6-*O*-(6-*O*-caffeoyl-β-d-glucopyranoside) (DPPH scavenging 89.1%) exhibited strong radical scavenging [22,23]. Additionally, quercetagetin-7-O-β-D-glucopyranoside showed vasorelaxation potential on PE (phenylephrine)-precontracted thoracic aortic rings in rats [24]. Tagenols A and B demonstrated potent lipoxygenase and antioxidant activities, with IC_50_ values of 12.2 μM and 5.8 μM, respectively, for lipoxygenase inhibition and IC_50_ values of 14.9 μM and 12.7 μM, respectively, for antioxidant capacity [25]. Quercetin 3-methyl ether exhibited various biological activities, including antioxidant, neuroprotective, anti-lipoxygenase, and anticancer activities [25,26,27]. Patuletin showed anti-arthritic and necrotic, antiproliferative, and apoptotic potentials in various tumor cell lines; however, its antidiabetic efficacy has not yet been explored [28,29].

Flavonols constitute a type of flavonoid that has been identified in almost all plants, including gymnosperms, angiosperms, mosses, ferns, and liverworts. As per estimates, over 1331 and 393 flavonol O-glycosides and flavonols have been characterized [30,31]. Flavonols accumulate in epidermal cells due to damage caused by pathogens, wounding, ozone irradiation, temperature variations, light, nutrient shortage, and co-pigmentation in fruits and flowers [30,32]. Preparations containing flavonol as a principal constituent have potential therapeutic or protective value against several human disorders, such as cardiovascular and neurodegenerative diseases, diabetes, platelet aggregation, and osteoporosis, among others [33,34,35]. Moreover, several studies have reported the alpha-amylase inhibitory potential of flavonoids or their synthetic analogs [36,37,38].

**Figure 1:**
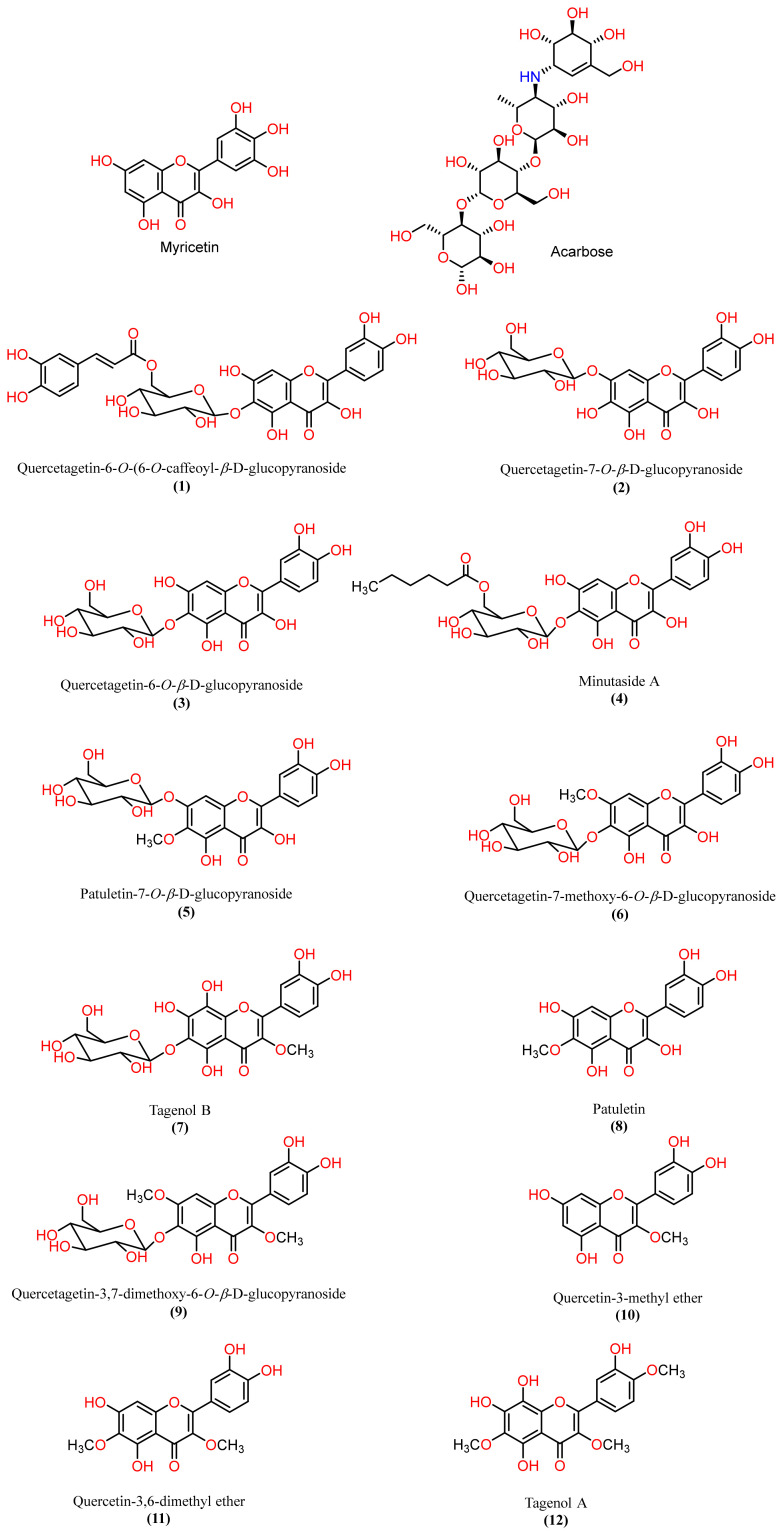
Chemical structures of the native inhibitor and the ligands of interest.

To further explore the AA inhibitory metabolites from natural sources, the flavonoids previously isolated by our group, namely, quercetagetin-6-O-(6-O-caffeoyl-β-D-glucopyranoside, quercetagetin-7-O-β-D-glucopyranoside, quercetagetin-6-O-β-D-glucopyranoside, minutaside A, patuletin-7-O-β-D-glucopyranoside, quercetagetin-7-methoxy-6-O-β-D-glucopyranoside, tagenols A and B, quercetagetin-3,7-dimethoxy-6-O-β-D-glucopyranoside, patuletin, quercetin-3,6-dimethyl ether, and quercetin-3-methyl ether, from *T. minuta* [6,22,25] were estimated for their in vitro AAI capacity (Figure 1).

Molecular docking is a bioinformatic tool that involves interactions between ligands and proteins. It is used to identify a target for a protein’s active site and to interpret the possible mechanism of activity. Ligands can form various interactions with proteins, including H-bonds, van der Waals forces, salt bridges, and hydrophobic bonds, which indicate their protein-binding tendency [39]. This tool is particularly useful in assessing the effectiveness of any metabolite prior to experimental investigation and represents a reliable and cost-efficient way to avoid random screening for bioactivities [40,41]. It is also a preliminary guide tool for discovering new drugs and has been reported to provide results comparable to those obtained from in vivo and in vitro studies. 

Therefore, in silico studies, such as molecular docking, dynamic simulation, and ADMET analyses, were performed for further validation of the in vitro AAI results of the tested flavonoids.

## 2. Results and Discussion

### 2.1. In Vitro AAI Activity

The discovery of the AAI potential of *T. minuta* flavonoids could provide evidence and ascertain the probable usage of *T. minuta* and its metabolites as alternative antidiabetic functional food for treating diabetes postprandial hyperglycemia. 

The in vitro AAI results (Table 1) revealed that all tested flavonoids exhibited AAI activities (IC_50s_ 7.8–26.9 µM). It is noteworthy that quercetagetin-7-O-β-D-glucopyranoside (**2**), quercetagetin-6-O-β-D-glucopyranoside (**3**), minutaside A (**4**), patuletin-7-O-β-D-glucopyranoside (**5**), and quercetagetin-7-methoxy-6-O- β-D-glucopyranoside (**6**) (IC_50_s 7.8–9.7 µM) had more potent AAI capacities comparable to that of acarbose (IC_50_ 7.1 µM). Additionally, quercetagetin-6-O-(6-O-caffeoyl-β-D-glucopyranoside (**1**), tagenols A (**12**) and B (**7**), quercetagetin-3,7-dimethoxy-6-O-β-D-glucopyranoside (**9**), patuletin (**8**), quercetin-3,6-dimethyl ether (**11**), and quercetin-3-methyl ether (**10**) demonstrated less effectiveness. It was noted that an increase in the number of OH groups increased AAI activity, while the replacement of the OH groups on the flavonoid core with OCH_3_ reduced the activity (e.g., **2** vs. **6** and **7** vs. **12**).

### 2.2. Molecular Docking Studies

After preparing the protein and ligands, their 3D structures were docked into the preselected grid box of pancreatic alpha-amylase (PDB ID: 4GQR). The selected 12 potential AAI compounds from *T. minuta* flavonoids, acarbose, and myricetin were included in our study (Figure 1, See Supplementary Materials Figures S1–S10). Subsequently, we utilized the LigPrep tools to prepare three-dimensional structures of these 14 compounds. The OPLS3 force field for energy minimization was employed during the preparation process. Moreover, Epik was utilized to generate all possible ionization states and tautomeric forms at a pH range of 7.0 ± 2 for each compound, and the corresponding hydrogen atoms were added accordingly. These preparatory steps generated 564 compounds, which were then subjected to our docking protocols. The docking scores were calculated using different methods, including Glide Emodel, Glide Gscore, and XP Gscore. The docking scores are listed in Table 1 based on the most negative XP scores. Our results show that acarbose, quercetagetin-7-O-β-D-glucopyranoside (**2**), quercetagetin-6-O-(6-O-caffeoyl-β-D-glucopyranoside (**1**), patuletin-7-O-β-D-glucopyranoside (**5**), quercetagetin-6-O-β-D-glucopyranoside (**3**), minutaside A (**4**), and quercetagetin-7-methoxy-6-O-β-D-glucopyranoside (**6**) had higher XP and Glide docking scores (−14.668 kcal/mol, −13.882 kcal/mol, −12.171 kcal/mol, −13.039 kcal/mol, −12.32 kcal/mol, −12.262 kcal/mol, and −12.22 kcal/mol, respectively) than the native inhibitor MYC (−11.723 kcal/mol). However, tagenol B (**7**), patuletin (**8**), quercetin-3-methyl ether (**10**), quercetagetin-3,7-dimethoxy-6-O-β-D-glucopyranoside (**9**), tagenol A (**12**), and quercetin-3,6-dimethyl ether (**11**) had docking scores lower than MYC (−11.277 kcal/mol, −9.013 kcal/mol, −8.163 kcal/mol, −9.445 kcal/mol, −8.69 kcal/mol, and −8.351 kcal/mol, respectively).

**Figure 2:**
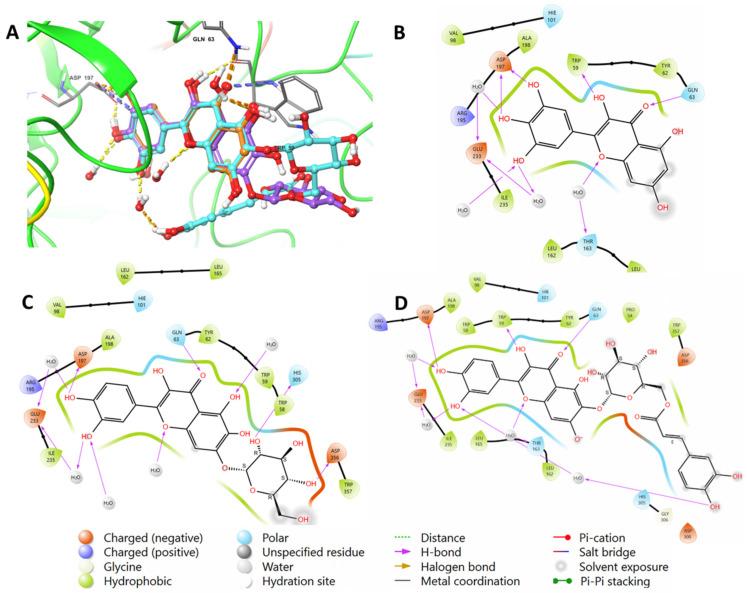
(**A**) A 3D view showing the superimposition of the native inhibitor MYC (orange), compound (**2**) (purple), and compound (**1**) (cyan) after docking. The 2D view shows interactions between pocket amino acids and MYC (**B**), compound (**2**) (**C**), and compound (**1**) (**D**).

To validate the findings, the native inhibitor MYC was redocked along with the other ligands, and the docking poses were examined. Figure 2 illustrates the binding interactions of the native inhibitor MYC with pancreatic alpha-amylase in both 3D and 2D views. Additionally, the top scoring compounds, namely, **1** and **2**, were superimposed on MYC. Notably, the MYC redocking revealed multiple hydrogen bonding interactions between the hydroxyl group, C=O, and oxygen in chromenone with Trp59 (2.45 Å), Gln63 (1.76 Å), and Thr163 through a water bridge. Additionally, there were three OH groups around the phenyl that interacted with Asp197 (1.81 Å and 1.64 Å) and Glu233 through water bridges. One aromatic hydrogen bond was observed between the C=O of chromenone and the phenyl part of Trp59. Furthermore, hydrophobic interactions were found around MYC through amino acids Trp59, Tyr62, Val98, Leu162, Lue165, Ala198, and Ile235. 

**Figure 3:**
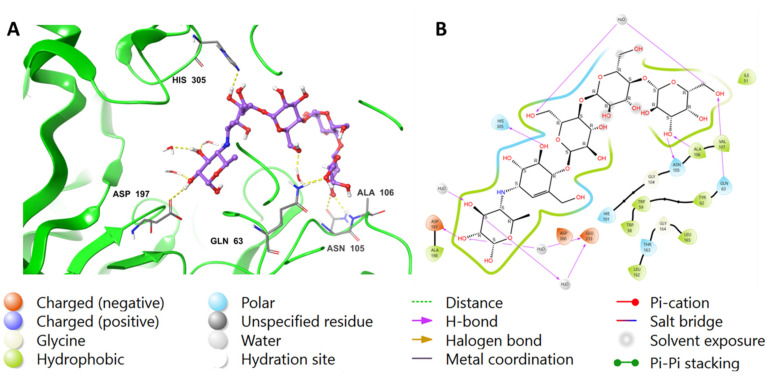
The putative binding mode of acarbose in the active site of pancreatic alpha-amylase (PDB ID: 4GQR). The purple sticks in the figure represent acarbose, while the yellow dotted lines represent hydrogen bonds, and the blue dotted lines represent ionic bonds. The figure is presented in two representations, a 3D and a 2D depiction, labeled as (**A**,**B**), respectively.

Similar to MYC, acarbose displayed H-bonds between its hydroxyl groups around the outer cyclohexane and Asp197 (2.55 Å), H_2_O (1.81 Å), and Glu233 via water bridges. Additionally, the cyclohexene moiety of acarbose was found to form an H-bond with His305 (2.07 Å). In the outer pyran ring, the hydroxyl group formed H-bonds with Asn105 (2.53 Å) and Ala106 (2.02 Å) amino acids, while the hydoxymethyl moiety formed H-bonds with H_2_O (2.62 Å) and Gln63 (1.87 Å). Furthermore, the hydoxymethyl group located in the middle pyran ring formed an H-bond with H_2_O (1.81 Å). The hydrophobic interactions involving acarbose included Ile51, Trp58, Trp59, Tyr62, Ala106, Val107, and Ala198 amino acids. The interactions are visualized in both 2D and 3D in Figure 3.

**Figure 4:**
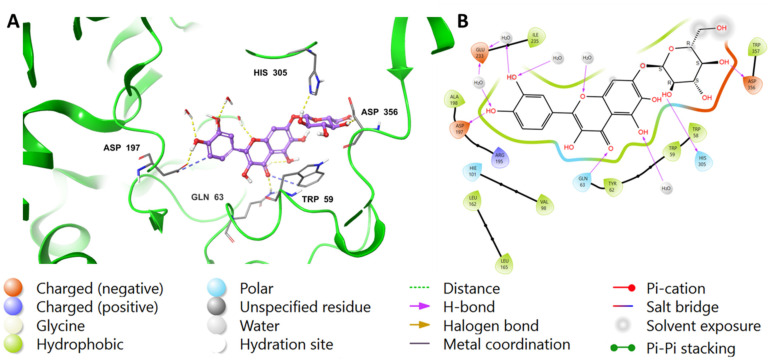
Binding mode of (**2**) in the active site of pancreatic alpha-amylase (PDB ID: 4GQR). Compound **2** is shown as purple sticks, while hydrogen bonds and ionic bonds are represented by yellow and blue dotted lines, respectively. (**A**) A 3D representation of pancreatic alpha-amylase complexed with (**2**), and (**B**) a 2D depiction.

**Figure 5:**
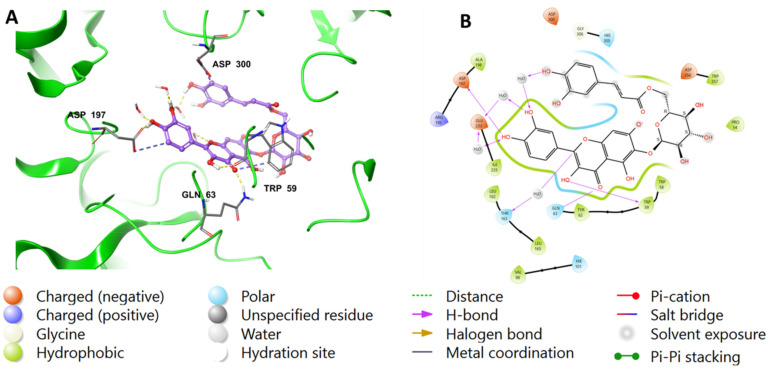
Binding mode of compound **1** in the active site of pancreatic alpha-amylase (PDB ID: 4GQR). Compound **1** appears as purple sticks in the figure, while the interactions, such as hydrogen bonds and ionic bonds, are depicted as yellow and blue dotted lines, respectively. (**A**) A 3D representation of pancreatic alpha-amylase complexed with (**1**), and (**B**) a 2D depiction.

The chromenone moiety of compounds **2** and **1** made intermolecular interactions with the pocket amino acids in a manner similar to that of the native inhibitor, MYC, which included H-bonding and water bridges, as well as some hydrophobic interactions. Additional interactions formed between the pyranose moieties of the compounds and the adjacent pockets. For compound **2**, the OH groups in the pyranose ring formed multiple H-bonds with Asp356 and His305 (Figure 4). The extended structure of **1** through the cinnamoyl moiety allowed it to form an aromatic H-bond with Asp300, as well as a water bridge between the phenolic OH and an adjacent water molecule (Figure 5). In addition to the list of interactions between the pancreatic alpha-amylase (PDB ID: 4GQR) and the rest of tested compounds in this study are mentioned in the Supplementary Materials Figures S1–S10.

The Prime/MM–GBSA equation was used to calculate the binding free energies (DGbind) of each ligand based on the docking complex. More negative values present a stronger binding (Table 1).

Proteins undergo movement in their backbone and sidechains when they bind to a ligand in a process known as induced-fit binding. This variability makes it difficult to assume a rigid receptor and to accurately model the binding process [42]. Therefore, induced-fit binding presents a challenging factor in drug design. The IFD scores of the best binding ligands are listed in Table 2. Our results demonstrate that all compounds had IFD scores comparable to those of the prepared ligand, indicating good binding. In fact, quercetagetin-6-O-(6-O-caffeoyl-β-D-glucopyranoside (**1**), quercetagetin-7-O-β-D-glucopyranoside (**2**), and quercetagetin-6-O-β-D-glucopyranoside (**3**) showed better IFD scores than the prepared ligand, indicating that these compounds have a higher likelihood of interacting well with the 4GQR receptor. 

### 2.3. QM/MM (Quantum Mechanics/Molecular Mechanics) Analysis

Quantum mechanics (QM) calculations can provide valuable insights into the electronic properties and interactions of protein–ligand complexes. However, due to the complexity and size of proteins, it is often necessary to use hybrid quantum mechanics/molecular mechanics (QM/MM) methods that combine a QM treatment of the ligand with a classical treatment of the protein and the solvent. The present study utilized an induced-fit docking (IFD) approach to identify the best pose of the molecule, which was then subjected to QM/MM computation. This method allowed for the treatment of the protein’s active site with the QM method, while the remainder of the protein was treated using the MM method. This approach has the advantage of providing accurate results while avoiding the high computational demands associated with calculating the QM for a large number of atoms [43]. The QM method further permits the precise computation of internal energies and HOMO/LUMO values [42], where HOMO and LUMO represent the highest occupied molecular orbital and the lowest unoccupied molecular orbital, respectively, located at the outer boundaries of the molecule’s electrons. The ionization potential is related to the HOMO, while the electron affinity is linked to the LUMO [44]. The energy difference between these two orbitals is referred to as the energy gap. In docking studies, the energy gap is used to estimate the binding affinity of the ligand to the protein. A smaller energy gap indicates a higher binding affinity and a stronger interaction between the protein and the ligand. 

In this study, the amino acids Gln63 and Asp197 were selected for the QM region, as they are common interaction sites for all the tested compounds. Table 2 presents the results of the QM/MM calculations, including the HOMO, LUMO, and binding energies for the 12 compounds. The compounds exhibit a range of HOMO and LUMO energies, with values between −0.068128 and −0.120463 kcal/mol and between 0.077919 and 0.148653 hartrees, respectively. The energy gaps, which are a measure of the stability of the compounds, range from 0.173 to 0.237 hartrees. The binding energies of the compounds were also calculated and ranged from −512.795165 to −512.687624 hartrees. The energy gaps suggest that the compounds are stable and may have potential as bioactive agents. However, further experiments are necessary to confirm their biological activity.

### 2.4. Molecular Dynamics Simulation

Molecular dynamics simulation is a reliable method used to evaluate and analyze the movements of atoms during a specific period of time in the system. Despite efforts to improve docking calculations and predictions, it still only provides a motionless perspective of a compound’s binding pose in the protein’s binding site [45]. Incorporating the classic equation of motion established by Newton [46], the MD simulation is used to study the dynamic behavior of protein–ligand complex stability in the system [46]. Desmond software (Version 6.0.129, Release 2022-4, Platform Linux-x86_64) was utilized to conduct MD studies on flavonoid inhibitors from *Tagetes minuta* with the best docking scores, the native inhibitor (myricetin), and acarbose (see Supplementary Materials Figures S11–S17). The MD simulation was run for a duration of 100 ns to ensure the convergence of pertinent system properties. 

Additionally, we performed a duplicate simulation to enable appropriate statistical analyses, and we provide the complete results for the MD simulation in the Supplementary Materials. The optimization of the complex structures at pH 7.0 2.0 followed a 100 ns simulation period. Predicting the stability of these structures involved analyzing interaction maps and the root mean square deviation plot of the protein and ligand [47].

**Figure 6:**
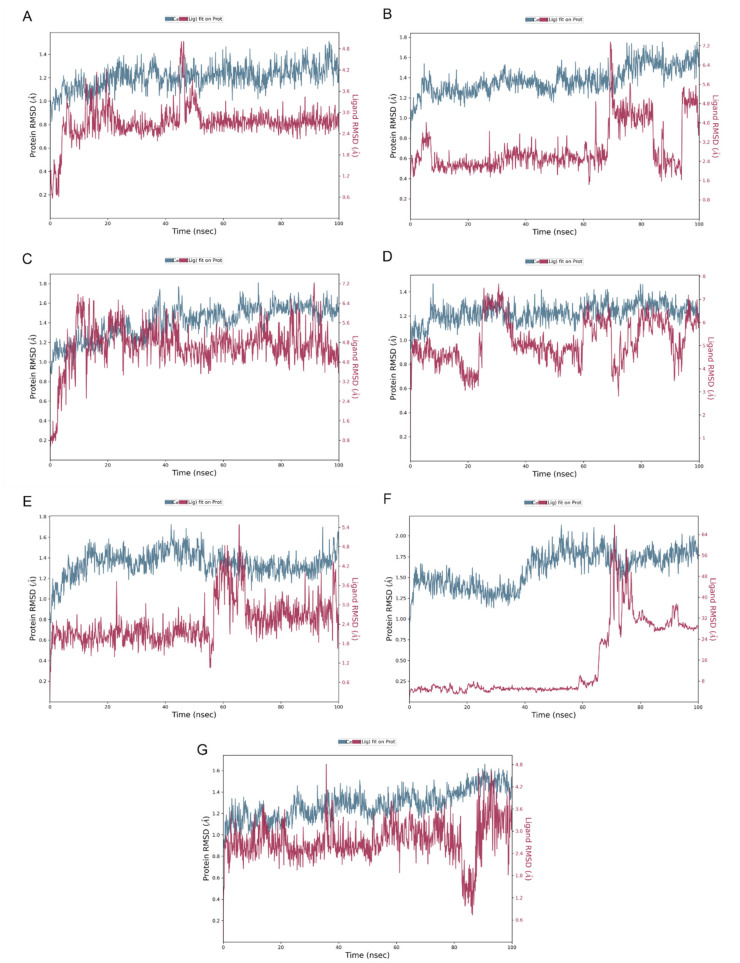
RMSD plot for (**A**) quercetagetin-7-O-β-D-glucopyranoside (**2**), (**B**) patu-letin-7-O-β-D-glucopyranoside (**5**), (**C**) 3-quercetagetin-6-O-β-D-glucopyranoside (**3**), (**D**) minutaside A (**4**), (**E**) quercetagetin-7-methoxy-6-O-β-D-glucopyranoside (**6**), (**F**) acarbose, (**G**) myricetin with pancreatic alpha-amylase (PDB ID: 4GQR) during the MD simulation.

Figure 6 illustrates the stability of the protein–ligand complexes during a 100 ns simulation with reference to the initial time point of 0 ns. The RMSD values of alpha-amylase and the ligands are plotted on the left and right y-axes, respectively. The quercetagetin-7-O-β-D-glucopyranoside (**2**), 3-quercetagetin-6-O-β-D-glucopyranoside (**3**), quercetagetin-7-methoxy-6-O-β-D-glucopyranoside (**6**), and myricetin complexes exhibited minor fluctuations within the acceptable range of 1–3 Å, indicating their stability. However, both patuletin-7-O-β-D-glucopyranoside (**5**) and minutaside A (**4**) displayed slight fluctuations beyond the acceptable range, up to 4 Å. The RMSD of acarbose revealed high fluctuations exceeding the acceptable range of 1–Å, indicating the instability of the alpha-amylase and acarbose complex and large conformational changes in the protein during the simulation. Overall, the data suggest that the majority of the complexes are stable, while the acarbose complex may require further investigation to improve its stability. Moreover, RMSD plot in duplicate for pancreatic alpha-amylase (PDB-ID: 4GQR) with the tested compounds during the MD simulation are represented in the Supplementary Materials Figures S11–S17.

**Figure 7:**
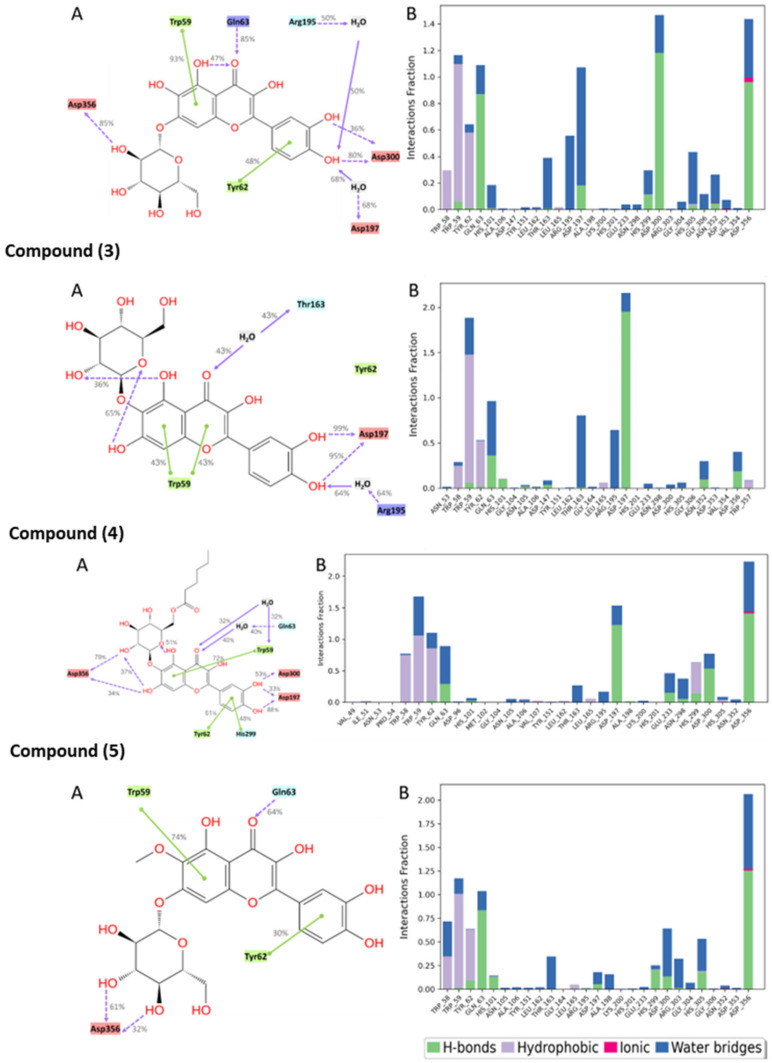
Interactions between the test compounds and pancreatic alpha-amylase (PDB ID: 4GQR) residues. Panel (**A**) depicts a detailed schematic representation of the interactions, where blue and green depict the polar and hydrophobic residues, respectively. Panel (**B**) displays a normalized stacked bar chart that represents the interaction between the receptor binding site residues and the compounds throughout the simulation.

**Figure 8:**
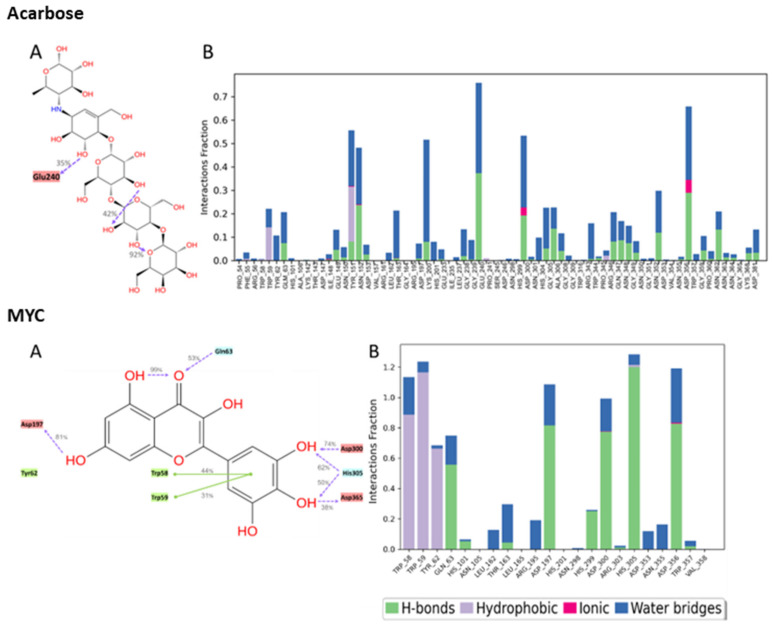
Interactions between acarbose and the native inhibitor MYC and pancreatic alpha-amylase (PDB ID: 4GQR) residues. Panel (**A**) depicts a detailed schematic representation of the interactions, where blue and green depict the polar and hydrophobic residues, respectively. Panel (**B**) displays a normalized stacked bar chart that represents the interaction between the receptor binding site residues and the compounds throughout the simulation.

The molecular interactions between the amino acid residues and the compounds, namely, quercetagetin-7-O-β-D-glucopyranoside (**2**), patuletin-7-O-β-D-glucopyranoside (**5**), 3-quercetagetin-6-O-β-D-glucopyranoside (**3**), minutaside A (**4**), acarbose, and MYC, are described in Figure 7 and Figure 8. The figures display the interactions that persisted for at least 30.0% of the simulation time within the selected frame (0.00 to 100.00 ns), as well as the docked poses that remained stable throughout the 100 ns simulation time. The different molecular interactions between the compounds and specific amino acid residues are presented as stacked bar charts that were normalized over the course of the trajectory. While a value of 1.0 (100%) indicates that the interaction was maintained throughout the 100.00 ns, a value > 100% reflects the involvement of more than one type of interaction during the simulation.

Multiple polar interactions formed between Asp356 and the pyranose OHs as well as the phenolic OHs and Asp300 of compound **2**, and a hydrophobic interaction formed between the chromenone and Trp59. Compound **5**′s most maintained interaction was with Asp356, while compound **3** showed stable interactions in the form of H-bonding with Asp197 and a hydrophobic interaction with Trp59, in addition to some water bridges. For compound **4**, Asp356, Asp197, and some hydrophobic interactions with Trp59 were detected. Acarbose, being a large molecule, expectedly formed many polar interactions with the pocket’s residues with values < 80%, which collectively contributed to its stabilization inside the pocket. For the native inhibitor, MYC, the main stabilizing interactions were with residues His305 and Trp59 and, to some extent, with Asp356, 300, and 197.

### 2.5. ADMET Properties

Table 3 presents a detailed analysis of the physicochemical properties and pharmacological activities of 14 compounds using various computational methods. The drug-likeness of each molecule is represented by the number of stars, with a higher number of stars indicating less drug-like properties. The number of reactive functional groups in each molecule was also determined to identify potential assay interference or toxicity. Furthermore, the CNS activity of each flavonoid was evaluated on a scale from −2 (inactive) to +2 (active). Other key properties, such as the molecular weight (mol_MW), total solvent-accessible surface area (SASA), estimated donor and acceptor hydrogen bonding with water molecules, predicted aqueous solubility (QPlogS), predicted octanol/water partition coefficient (QPlogPo/w), prediction of binding to human serum albumin (QPlogkhsa), predicted brain/blood partition coefficient (QplogBB), number of likely metabolic reactions (#metab), predicted human oral absorption (PercentHumanOralAbsorption), and predicted IC_50_ values for the blockage of HERG K+ channels (QPlogHERG), were also determined. Among the analyzed flavonoids, acarbose showed the highest number of stars, suggesting that it is less drug-like than the other compounds. 

Acarbose, a complex oligosaccharide, exhibits a competitive and reversible inhibition of pancreatic alpha-amylase and membrane-bound intestinal alpha-glucoside hydrolase. Its primary site of action is within the gastrointestinal tract, specifically targeting the surface of enterocytes. Notably, acarbose demonstrates minimal systemic absorption, with a mere 2% of the oral dose absorbed into the bloodstream. Most acarbose metabolism occurs within the gastrointestinal tract, facilitated by intestinal bacteria and digestive enzymes. Moreover, renal excretion significantly eliminates the drug, accounting for approximately 51% of an oral dose through fecal excretion. It is worth mentioning that Table 3 highlights the similarity in pharmacokinetic parameters, target selectivity, limited oral absorption, and cell permeability among the tested compounds, including acarbose.

## 3. Materials and Methods

### 3.1. AAI Evaluation 

The AAI capacity of *T. minuta* flavonoids was estimated using an Ultra-Amylase^®^Enz-Chek Assay Kit in line with a formerly reported method [48,49].

### 3.2. Preparation of Proteins

The crystal structure of human pancreatic alpha-amylase in complex with myricetin (MYC) was obtained from the Protein Data Bank (PDB ID: 4GQR) [50]. The protein was prepared for further analysis using the Protein Preparation Wizard in Maestro Schrödinger [51]. This included the addition of missing hydrogen atoms to the residues, the correction of metal ionization states, and the removal of water molecules beyond 5 Å. The option to keep water molecules within a certain distance (typically 5 Å) around the ligand was available to account for possible water-mediated interactions and solvation effects. Additionally, the protein was assigned appropriate charges, and restrained minimization was performed with the OPLS4 force field.

Myricetin is a member of the flavonol family, and it is derived from various vegetables and fruits. It was proved to possess antioxidant and α-amylase and α-glucosidase inhibitory potential [52]. Additionally, a cross-sectional population study by Yao et al. revealed that myricetin intake had a protective effect in type II DM development in a Chinese population [53].

### 3.3. Ligand Preparation 

In this study, we utilized Schrödinger’s LigPrep tool [54] to dock 12 potential AAI compounds from Tagetes Flavonoids and included acarbose and myricetin in our study. Subsequently, we utilized the LigPrep tools to prepare three-dimensional structures of these 14 compounds. We employed the OPLS3 force field for energy minimization during the preparation process. Moreover, Epik was employed to generate all possible ionization states and tautomeric forms at a pH range of 7.0 ± 2 for each compound, and the corresponding hydrogen atoms were added accordingly. Furthermore, PROPKA was used to optimize hydrogen bonds, and any water molecules located beyond 3 Å away from the HET groups were removed from consideration. This ensured the accuracy and reliability of our results while minimizing any potential sources of error. These preparatory steps generated 564 compounds, which were then subjected to our docking protocols.

### 3.4. Grid Generation and Molecular Docking

**Figure 9:**
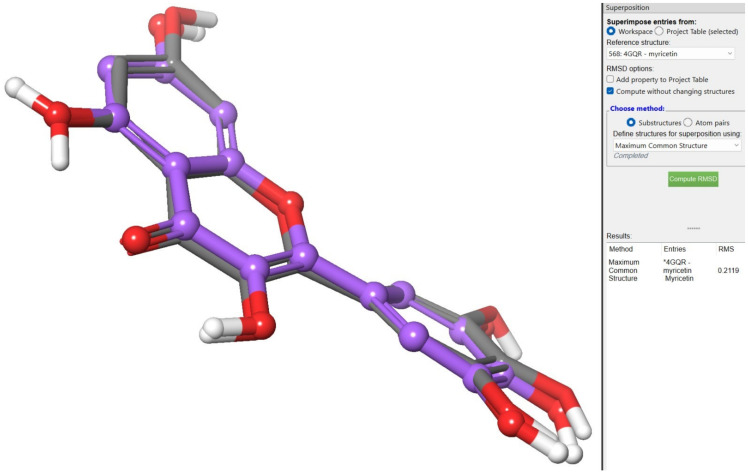
Redocking experiments to validate docking accuracy. Co-crystallized ligand (MYC)* was redocked into the prepared protein, comparing predicted binding pose (blue) with crystallographic pose (green) of MYC.

In the co-crystallized structure of 4GQR, the ligand MYC was chosen as the reference for defining the grid box, and Glide’s Receptor-Grid-Generation tool was employed to specify the binding site [55]. The grid box dimensions were set to 10 Å in each of the X, Y, and Z directions, using the default settings without any modifications. Ligand docking was performed using the Ligand Docking Tool in the Schrödinger suite, employing the extra-precision (XP) protocol with a partial charge cut-off of 0.25 and a van der Waals radius scaling factor of 1.0. To validate the accuracy of the docking method, redocking experiments were conducted by docking the co-crystallized ligand (MYC) back into the prepared protein. The main objective was to assess the accuracy of the predicted binding pose by comparing it with the crystallographic pose of MYC. Surprisingly, the predicted binding pose closely resembled the crystallographic pose, exhibiting a remarkably low root mean square deviation (RMSD) value of 0.2119. This finding strongly indicates a high level of agreement between the predicted and observed binding interactions of MYC (Figure 9).

For further validation of the docking method, redocking experiments were performed with the co-crystallized ligand and the compounds of interest. The docking scores were evaluated using the G score, which ranks the compounds. GlideScore, an empirical scoring function designed to estimate the binding free energy of ligands, was employed to approximate the ligand binding free energy and to rank the different ligand poses. GlideScore incorporates various terms, including contributions from force fields (such as electrostatic and van der Waals forces) and terms that account for interactions known to influence ligand binding. The scoring function has been optimized to accurately predict docking, enrich databases, and predict binding affinity. In virtual screening, more negative GlideScore values indicate tighter binding. It is important to note that the XP GlideScore shares common terms with the SP and HTVS GlideScore, but they have been optimized separately and cannot be directly compared [56]. Emodel, which focuses on force field components (electrostatic and van der Waals energies), is particularly suitable for comparing conformers but less effective for comparing chemically distinct species. Therefore, Glide utilizes Emodel to select the best pose of a ligand, and then it employs GlideScore to rank these selected poses against each other. In the case of Glide XP, the pose selection procedure is slightly more complex and involves Emodel and GlideScore. Poses generated by Glide XP are assigned an “XP PoseRank” property, indicating their ranking for a given ligand according to Glide XP. Additionally, the XP Gscore was used to rank the ligands’ ability to bind to a specific conformation of the protein receptor, following the corresponding equation:

XP GlideScore = Ecoul + EVdW + Ebind + Epenalty

Ebind = E*_hyd_enclosure_* + E*_hb_nn_motif_* + E*_hb_cc_motif_* + E*_PI_* + E*_hb_pair_* + E*_phobic_pair_*

Epenalty = E*_desolv_* + E*_ligand_strain_*

where E is the energy, E*_coul_* is the Coulomb energy, E*_VdW_* is the van der Waals energy, E*_bind_* is the energy that promotes binding, and E*_penalty_* is the energy that impedes binding. The XP GlideScore was calculated using various descriptors, including E*_hyd_enclosure_* (the hydrophobic enclosure), E*_hb_nn_motif_* (special neutral–neutral hydrogen bond motifs), E*_hb_cc_motif_* (special charged–charged hydrogen bond motifs), E*_PI_* (pi-cation interactions), E*_hb_pair_* (hydrogen bond pair), E*_phobic_pair_* (lipophilic pair), and E*_desolv_* (desolvation energy) [57]. MM-GBSA (molecular mechanics–generalized born surface area) calculations were carried out using Prime for re-scoring the docked poses. The binding free energy was calculated using the following equation:

ΔGbind = E_Complex (minimized) − E_ligand (minimized) − E_protein (minimized)

where (Complex) is the protein–ligand complex (Eprotein).

### 3.5. Induced-Fit Docking

The induced-fit docking (IFD) method, which models the conformational changes induced by ligand binding, was developed by Schrödinger [58]. In this approach, the complex is used to generate the centroid of the residues by selecting the ligand from the protein. The softened potential (van der Waals radius scaling) is initially used to perform the docking of each ligand. Following this, sidechain prediction is undertaken within a particular distance of the ligand pose. Next, a minimization step is performed for the same set of residues as well as the ligand for each protein–ligand complex pose. Consequently, the receptor structure in each pose represents an induced fit to the ligand structure and conformation. Eventually, the ligand is docked more rigorously into the induced-fit receptor structure using Glide XP. During the initial docking, both the receptor and ligand were subjected to a van der Waals scaling factor of 0.5. The Prime refinement step was applied to the sidechains of the residues within 5 Å of the ligand. A maximum of 20 poses was retained for each ligand docked for further redocking in XP mode.

### 3.6. Molecular Dynamics Simulation (MD)

To perform the molecular dynamics simulation, we utilized the Desmond software from the Schrödinger package version 13.5.128 [59], limiting the run to only include the compounds with the highest docking scores. To ensure accurate results, we immersed the protein–ligand complex into a solvated system, which was created by placing the complex into an orthorhombic water box that extended 10 Å beyond the atoms in the complex. To neutralize the system, we added Na and Cl counter ions. The simulation was set to continue for 100 ns, maintaining a constant temperature of 300 K and a constant pressure of 1.01325 bar. 

### 3.7. QM/MM (Quantum Mechanics/Molecular Mechanics) Calculations

The geometries utilized in the QM/MM calculations were obtained from the induced-fit docking process described earlier. The QM/MM calculations were conducted on the protein with PDB-ID 4GQR, together with the native inhibitor (PDB-ID: MYC) and the ligands that had the highest docking scores. To determine the QM/MM, the Q site program [60,61] was employed. This entailed including the ligand in addition to the interacting residues. The QM region was constructed by selecting the free ligand and its sidechains. For QM calculations, we selected the DFT-B3LP method, which is a function of electron density. The QM system had a charge of -1. The MM region, which consisted of the rest of the system, was treated with the OPLS-2005 force field. Upon the completion of the QM/MM run, we calculated the HOMO (highest occupied molecular orbital), LUMO (lowest unoccupied molecular orbital), and energy gaps.

### 3.8. Prediction of ADMET

Predicting the ADME properties of the compounds, which are absorption, distribution, metabolism, and elimination, provides some vital information related to the drugs/molecules. All compounds were assessed using the Maestro Schrodinger QikProp module to identify promising molecules following bioavailability property and ADMET profiling [62].

## 4. Conclusions

Diabetes is a lifestyle-induced metabolic disorder differentiated by chronic hyperglycemia, dyslipidemia, and impeded protein metabolism resulting in multi-organ injury. AAI controls postprandial hyperglycemia via the slowdown or deactivation of starch breakdown. In vitro and molecular docking investigations revealed that the isolated *T. minuta* flavonoids notably prohibited the AA enzyme. Hence, the current study highlights the possible usage of *T. minuta* as a functional food. Moreover, this could provide an avenue for further examination of these metabolites as potential AAI antidiabetics for treating diabetes. However, extra experimental studies are required to affirm the findings obtained in this work.

The present study investigated the inhibitory activity of natural flavonoids reported from *T. minuta* against alpha-amylase. These metabolites demonstrated alpha-amylase inhibition capacities in an in vitro assay. Further, docking studies using the Schrodinger suite of software tools version 13.5.128 were employed to identify the binding affinity of these ligands towards the receptor, and molecular dynamics simulations were carried out using the IFD protocol to validate the in vitro results. The generated results show that quercetagetin-7-O-β-D-glucopyranoside (**2**) and acarbose had the highest binding energies with the receptor. The MMGBSA dG bind values showed a clear contrast between acarbose and quercetagetin-7-O-β-D-glucopyranoside (**2**), with the former exhibiting a much stronger stabilization energy. The computational studies’ results are in good agreement with those of the in vitro data. These findings revealed that quercetagetin-7-O-β-D-glucopyranoside (**2**) might be a promising lead for new alpha-amylase inhibitors. However, further mechanistic and in vivo studies are needed.

## Figures and Tables

**Table 1:** Docking results of in silico screening for the compounds based on their XP Gscores against pancreatic alpha-amylase receptor (PDB ID: 4GQR), MMGBSA, and in vitro AAI assay.

	Docking Scores				MMGBSA		AAI
Compound	Docking Score (kcal/mol)	XP Gscore (kcal/mol)	Glide Gscore (kcal/mol)	Glide Emodel (kcal/mol)	Prime Energy (kcal/mol)	dG Bind	IC_50_ μM
**Acarbose**	−14.668	−15.22	−15.22	−79.824	−20,790.9	−33.97	7.1
**2**	−13.882	−13.889	−13.889	−87.396	−21,006.9	−51.04	7.8
**1**	−12.171	−13.868	−13.868	−97.793	−21,071.5	−17.09	10.1
**5**	−13.039	−13.039	−13.039	−85.651	−21,027.7	−48.4	8.1
**3**	−12.32	−12.362	−12.362	−89.862	−21,074.6	−54.74	8.9
**4**	−12.262	−12.305	−12.305	−91.452	−21,096.1	−64.34	9.2
**6**	−12.22	−12.22	−12.22	−88.197	−21,043.3	−55.43	9.7
**Myricetin (MYC)**	−11.723	−11.77	−11.77	−65.617	−21,172.2	−43.5	−
**7**	−11.277	−11.323	−11.323	−74.269	−20,984	−38.32	10.8
**8**	−9.013	−10.771	−10.771	−59.642	−21,093.4	−19.84	13.6
**10**	−8.163	−9.874	−9.874	−50.031	−21,155.8	−7.33	26.9
**9**	−9.445	−9.445	−9.445	−73.229	−21,043.5	−36.31	12.7
**12**	−8.69	−8.727	−8.727	−65.035	−21,020.6	−42.06	14.9
**11**	−8.351	−8.392	−8.392	−56.918	−21,115.6	−33.39	21.7

**Table 2:** IFD Score, HOMO, LUMO, and binding energies (hartrees) for test compounds.

Compound	IFD Score	Number of Canonical Orbitals	QM/MM Energy	HOMO	LUMO	Energy Gap
**Acarbose**	−1131.85	170	−512.728183	−0.108797	0.091728	0.201
**2**	−1133.46	170	−512.694847	−0.10958	0.088141	0.198
**1**	−1134.96	170	−512.786174	−0.068128	0.121556	0.190
**5**	−1131.74	170	−512.708656	−0.103067	0.088501	0.192
**3**	−1133.32	170	−512.782343	−0.118446	0.081654	0.200
**4**	−1132.87	170	−512.795165	−0.094984	0.077919	0.173
**6**	−1131.09	170	−512.751505	−0.108385	0.103096	0.211
**Myricetin (MYC)**	−1132.91	170	−512.7611	−0.118972	0.093787	0.213
**7**	−1131.83	170	−512.775685	−0.098428	0.099785	0.198
**8**	−1130.88	170	−512.753084	−0.106348	0.083171	0.190
**10**	−1129.54	170	−512.687624	−0.08808	0.148653	0.237
**9**	−1131.07	170	−512.787028	−0.11625	0.098969	0.215
**12**	−1127.55	170	−512.724293	−0.118931	0.090068	0.209
**11**	−1130.52	170	−512.771419	−0.120463	0.098182	0.219

HUMO: highest occupied molecular orbital; QM/MM: quantum mechanism/molecular mechanics; LUMO: lowest unoccupied molecular orbital.

**Table 3:** In silico ADME properties of tested depsidone derivatives.

Molecule	mol_MW	# Stars	# rtvFG	CNS	SASA	donorHB	accptHB	QPlogPo/w	QPlogHERG	QPPCaco	QPlogBB	# Metab	QPlogKhsa	Percent Human Oral Absorption
Recommended Range	(130–725)	(0.0–5.0)	(0–2)	(−2 Inactive) (+2 Active)	(300–1000)	(0–6)	(2.0–20.0)	(−2–6.5)	Concern Below −5	<25 Poor, >500 Great	(−3–1.2)	(1–8)	(−1.5–1.5)	(<25% Poor; >80% High)
**Acarbose**	807.753	15	4	−2	992.409	17	40.6	−9.2	−5.826	0.012	−6.846	17	−3.359	0
**2**	480.381	5	1	−2	683.765	8	14.5	−2.0	−5.313	1.608	−4.141	9	−1.036	0
**5**	494.408	5	1	−2	711.037	7	14.5	−1.4	−5.427	2.135	−4.066	9	−0.934	0
**3**	480.381	5	1	−2	706.734	8	14.5	−2.0	−5.561	1.002	−4.503	9	−1.033	0
**4**	578.526	8	2	−2	887.136	7	14.8	0.3	−6.362	3.315	−4.673	9	−0.656	0
**6**	494.408	5	1	−2	719.054	7	14.5	−1.3	−5.397	2.744	−3.978	9	−0.917	1.3
**1**	642.526	8	2	−2	813.947	9	16.3	−1.1	−5.482	0.318	−5.127	10	−0.79	0
**Myricetin**	318.239	0	0	−2	517.206	5	6	−0.3	−4.756	7.701	−2.778	6	−0.496	28.1
**7**	510.407	5	1	−2	735.455	8	15.25	−1.7	−5.474	1.863	−4.3	10	−1.043	0
**8**	332.266	0	0	−2	543.866	4	6	0.7	−4.906	33.094	−2.215	6	−0.317	58.0
**9**	508.435	4	1	−2	737.814	6	14.5	−0.6	−5.321	6.203	−3.595	9	−0.805	0
**12**	376.319	0	0	−2	643.535	3	6.75	1.9	−5.595	105.079	−2.005	7	−0.038	74.3
**11**	346.293	0	0	−2	570.127	3	6	1.5	−4.962	89.029	−1.826	6	−0.133	70.4
**10**	316.267	0	0	−2	521.456	3	5.25	1.1	−4.725	59.31	−1.826	5	−0.182	65.2

**Abbreviations:** molecular weight (mol_MW), drug-likeness (# Stars), total solvent-accessible surface area (SASA), number of hydrogen bond donors and acceptors (donorHB and acceptHB), predicted octanol/water partitioning (QPlogPo/w), estimated binding to human serum albumin (QPlogKhsa), number of possible metabolites (# Metab), predicted blood/brain partitioning (QPlogBB), percentage of human oral absorption, predicted IC50 for inhibiting HERG-K+ channels (QPogHERG), predicted apparent Caco-2 cell permeability in nm/sec for gut–blood barrier (QPPCaco), central nervous system activity (CNS), number of reactive functional groups present (# rtvFG), and percent human oral absorption.

## Data Availability

Data is contained within the article and Supplementary Materials.

## Supplementary Materials

The supplementary information for this study is available for download at the following location: https://www.mdpi.com/article/10.3390/ijms241210195/s1.
