# Supplementary material for: Assessments of Alpha-Amylase Inhibitory Potential of Tagetes Flavonoids through In Vitro, Molecular Docking, and Molecular Dynamics Simulation Studies
Source: Int J Mol Sci. 2023 Jun 15;24(12):10195. doi: 10.3390/ijms241210195 (PMC10298979; doi:10.3390/ijms241210195)
Supplement: Supplementary file 1 [file ijms-24-10195-s001.zip › ijms-2431480-supplementary.pdf]

International Journal of *Molecular Sciences*

MDPI

Supplementary Materials

Assessments of Alpha-amylase Inhibitory Potential of Tagetes Flavonoids through In-vitro, Molecular Docking, and Molecular Dynamic Simulation Studies

Gamal A. Mohamed [1,*], Abdelsattar M. Omar [2,3], Moustafa E. El-Araby [2], Shaza Mass [2], Sabrin R. M. Ibrahim [4,5]

[1] Department of Natural Products and Alternative Medicine, Faculty of Pharmacy, King Abdulaziz University, Jeddah 21589, Saudi Arabia

[2] Department of Pharmaceutical Chemistry, Faculty of Pharmacy, King Abdulaziz University, Jeddah 21589, Saudi Arabia; asmansour@kau.edu.sa (A.M.O.); madaoud@kau.edu.sa (M.E.E.); shazaamass@gmail.com (S.M.)

[3] Center for Artificial Intelligence in Precision Medicines, King Abdulaziz University, Jeddah 21589, Saudi Arabia

[4] Preparatory Year Program, Department of Chemistry, Batterjee Medical College, Jeddah 21442, Saudi Arabia; sabrin.ibrahim@bmc.edu.sa

[5] Department of Pharmacognosy, Faculty of Pharmacy, Assiut University, Assiut 71526, Egypt

* Correspondence: gahussein@kau.edu.sa

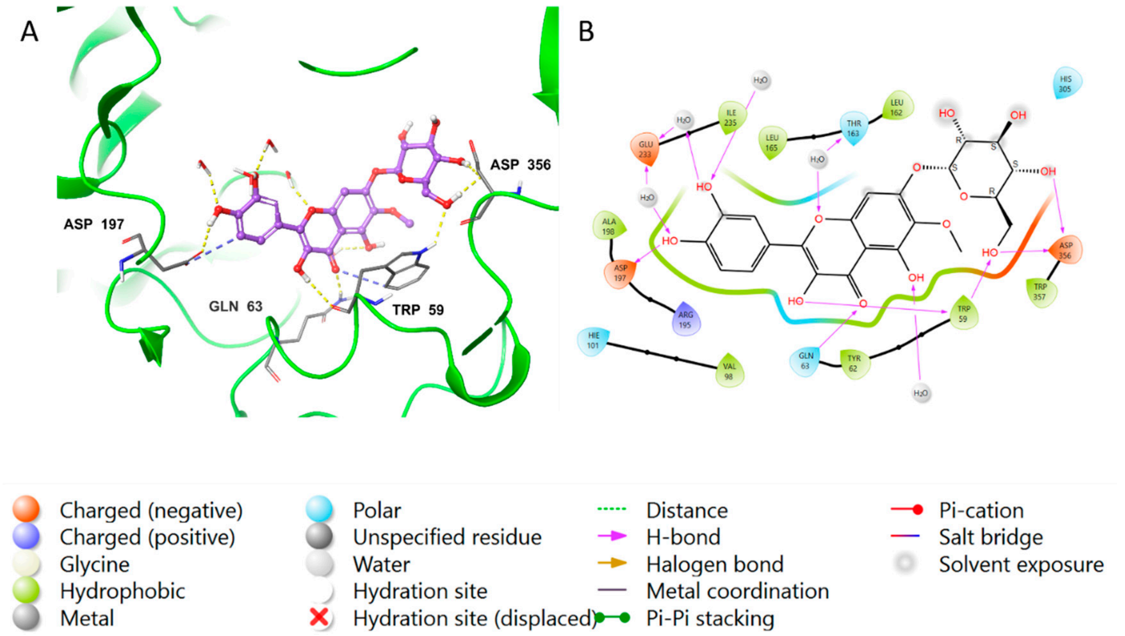

Figure S1. Proposed binding mode of patuletin-7-O-β-D-glucopyranoside (**5**) in the active site of the pancreatic alpha-amylase (PDB ID: 4GQR) in both 3D (**A**) and 2D (**B**) views.

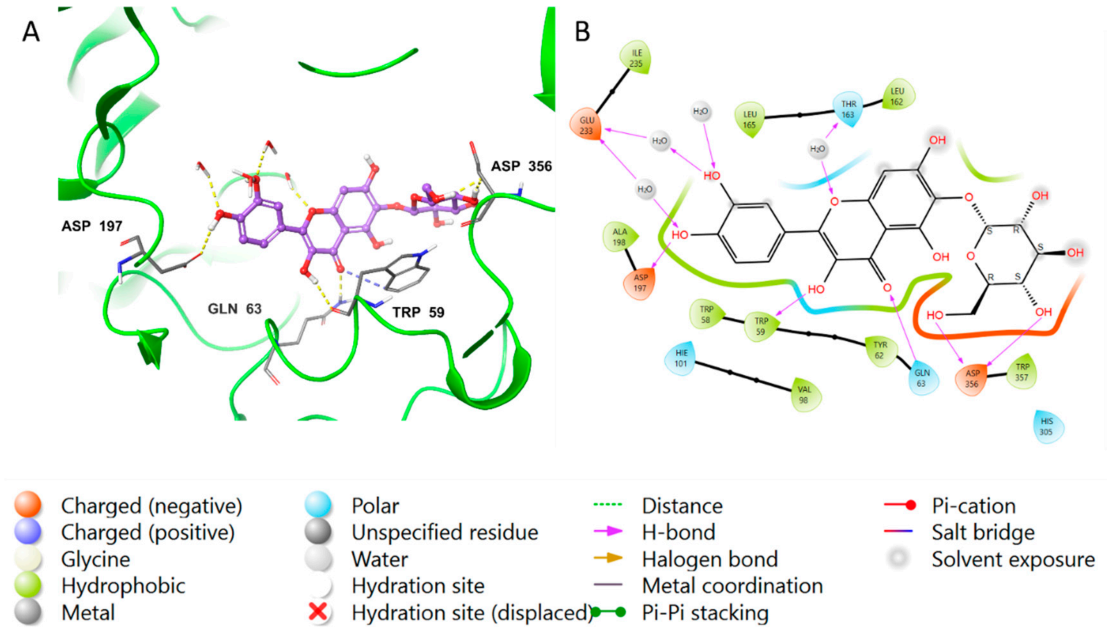

Figure S2. Proposed binding mode of quercetagetin-6-O-β-D-glucopyranoside (**3**) in the active site of the pancreatic alpha-amylase (PDB ID: 4GQR) in both 3D (**A**) and 2D (**B**) views.

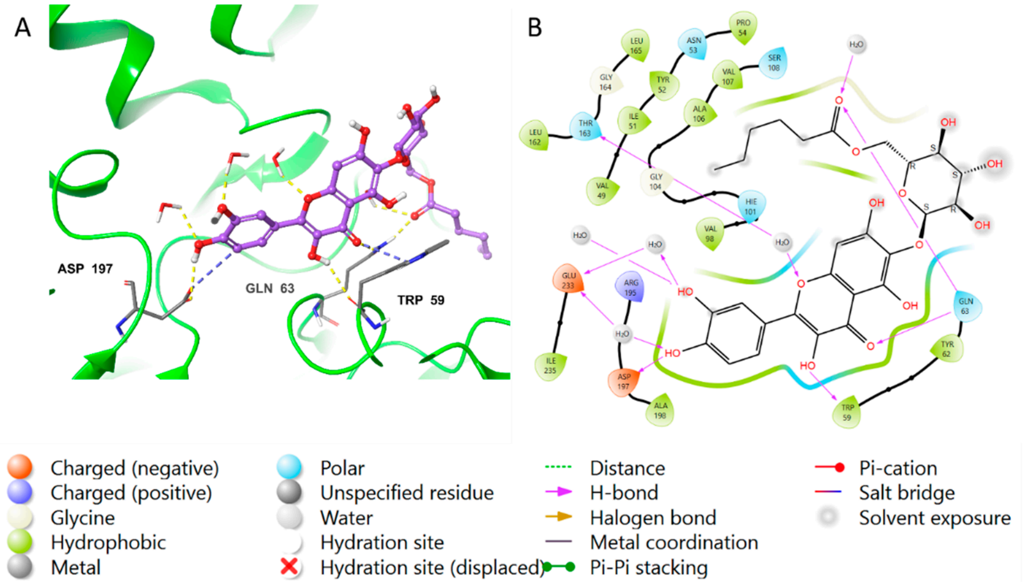

Figure S3. Proposed binding mode of minutaside A (**4**) in the active site of the pancreatic alpha-amylase (PDB ID: 4GQR) in both 3D (**A**) and 2D (**B**) views.

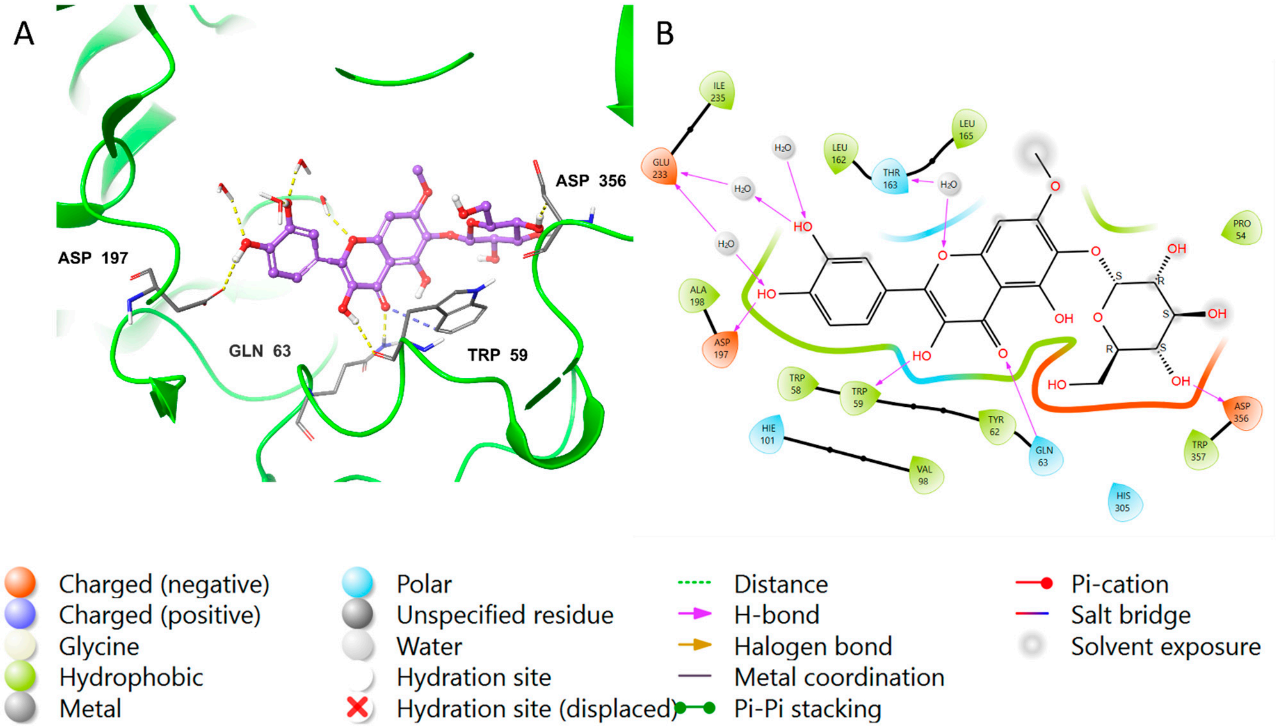

Figure S4. Proposed binding mode of quercetagetin-7-methoxy-6-O-β-D-glucopyranoside (**6**) in the active site of the pancreatic alpha-amylase (PDB ID: 4GQR) in both 3D (**A**) and 2D (**B**) views.

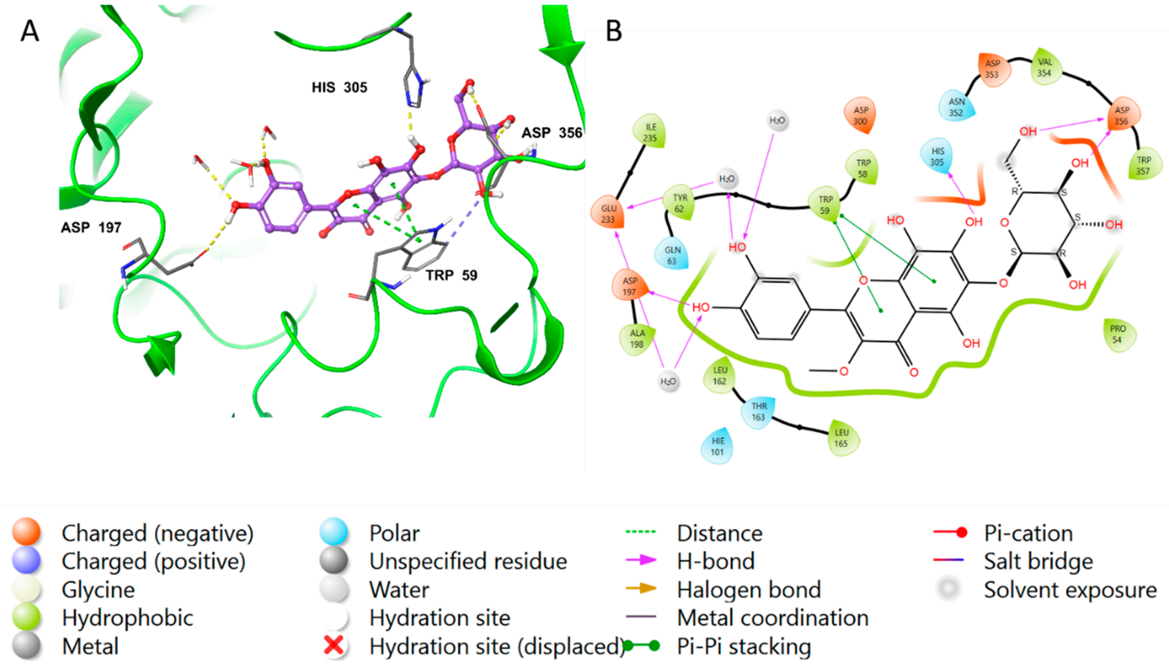

Figure S5. Proposed binding mode of tagenol B (**7**) in the active site of the pancreatic alpha-amylase (PDB ID: 4GQR) in both 3D (**A**) and 2D (**B**) views.

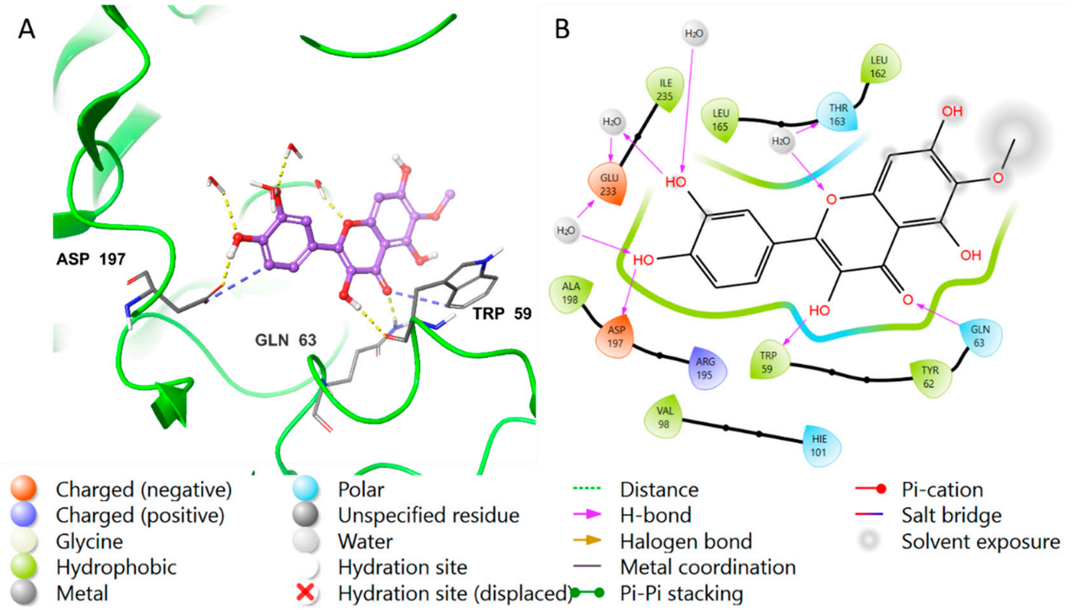

Figure S6. Proposed binding mode of patuletin (**8**) in the active site of the pancreatic alpha-amylase (PDB ID: 4GQR) in both 3D (**A**) and 2D (**B**) views.

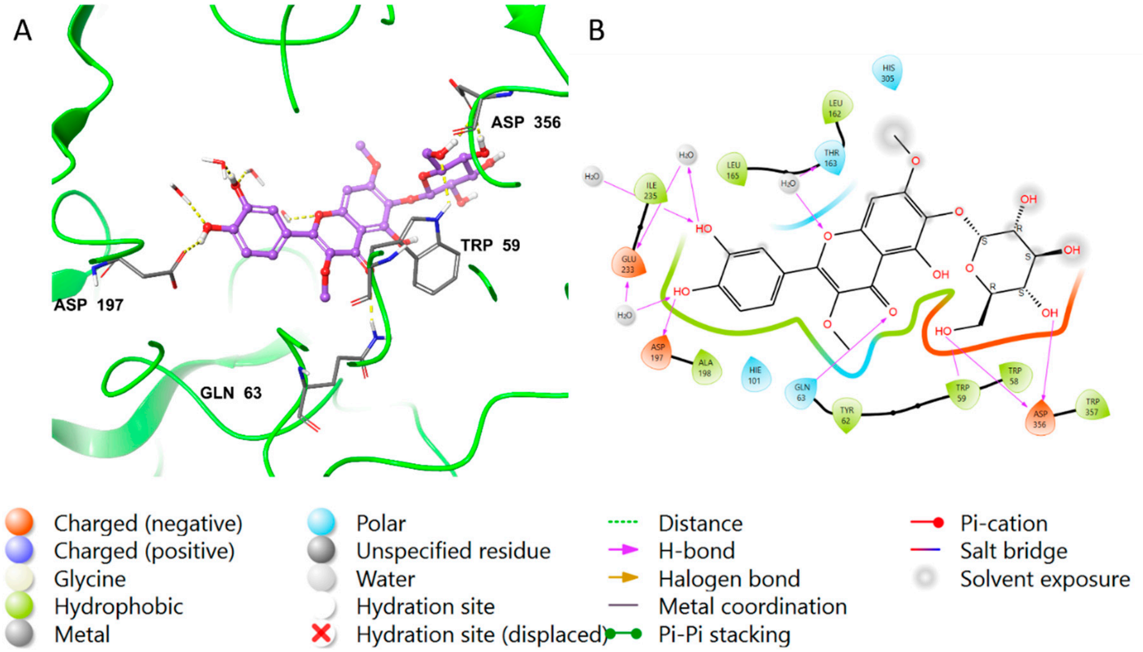

Figure S7. Proposed binding mode of quercetagetin-3,7-dimethoxy-6-O-β-D-glucopyranoside (**9**) in the active site of the pancreatic alpha-amylase (PDB ID: 4GQR) in both 3D (**A**) and 2D (**B**) views.

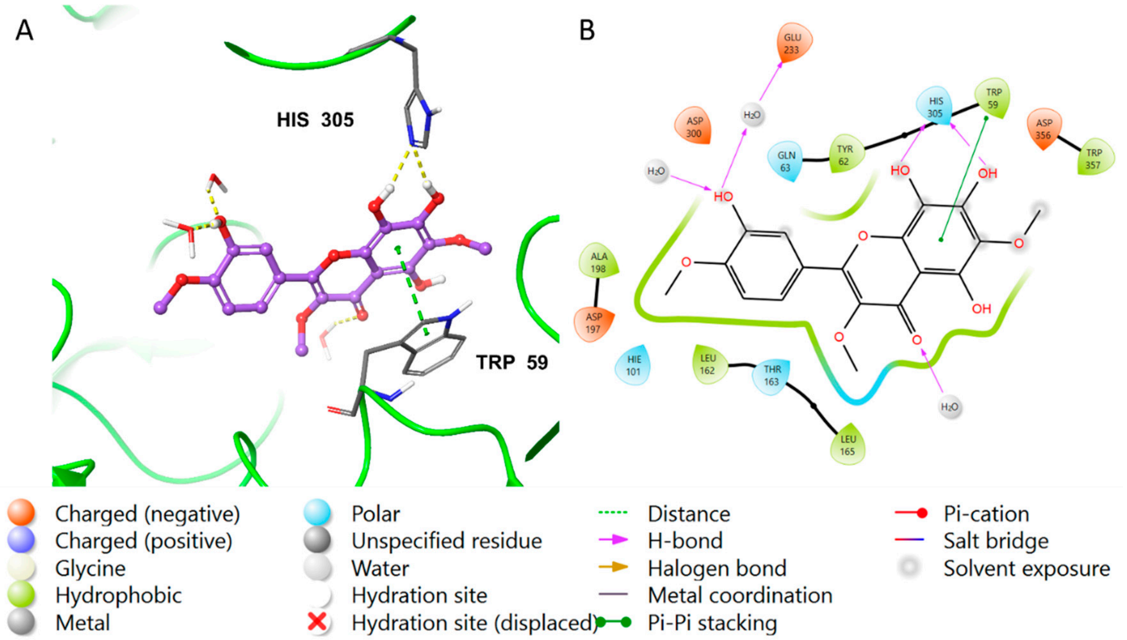

Figure S8. Proposed binding mode of tagenol A (**12**) in the active site of the pancreatic alpha-amylase (PDB ID: 4GQR) in both 3D (**A**) and 2D (**B**) views.

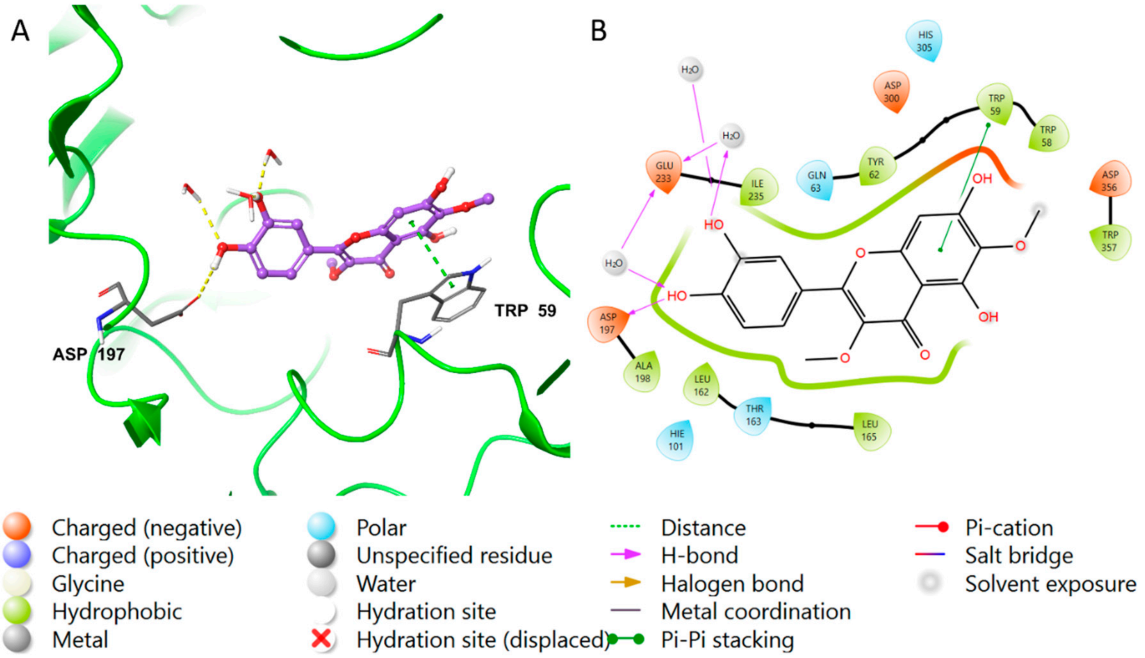

Figure S9. Proposed binding mode of quercetin-3,6-dimethyl ether (**11**) in the active site of the pancreatic alpha-amylase (PDB ID: 4GQR) in both 3D (**A**) and 2D (**B**) views.

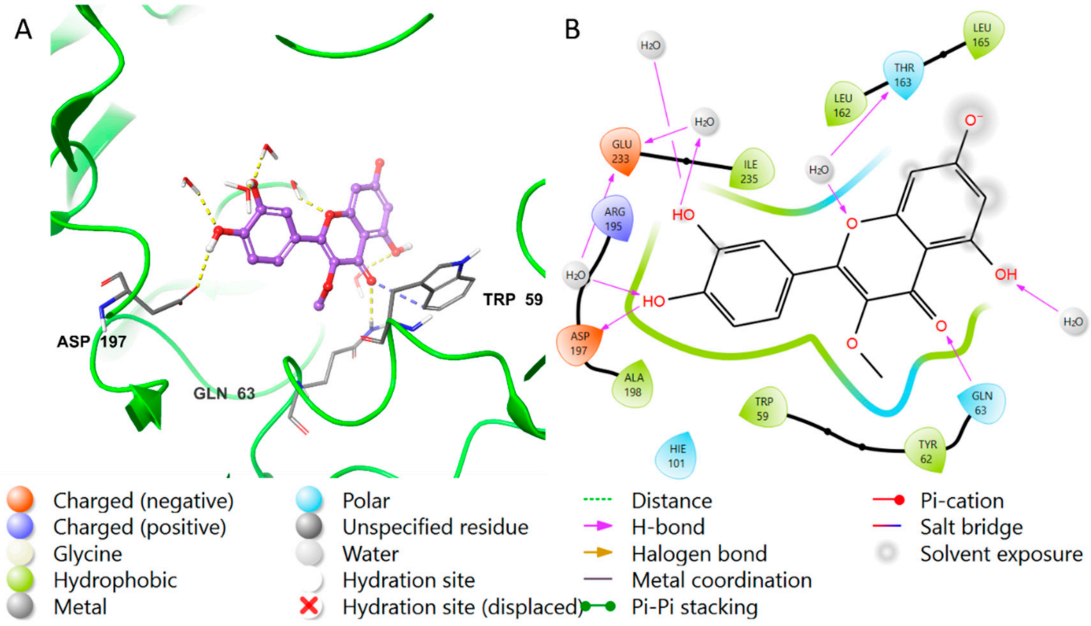

Figure S10. Proposed binding mode of quercetin-3-methyl ether (**10**) in the active site of the pancreatic alpha-amylase (PDB ID: 4GQR) in both 3D (**A**) and 2D (**B**) views.

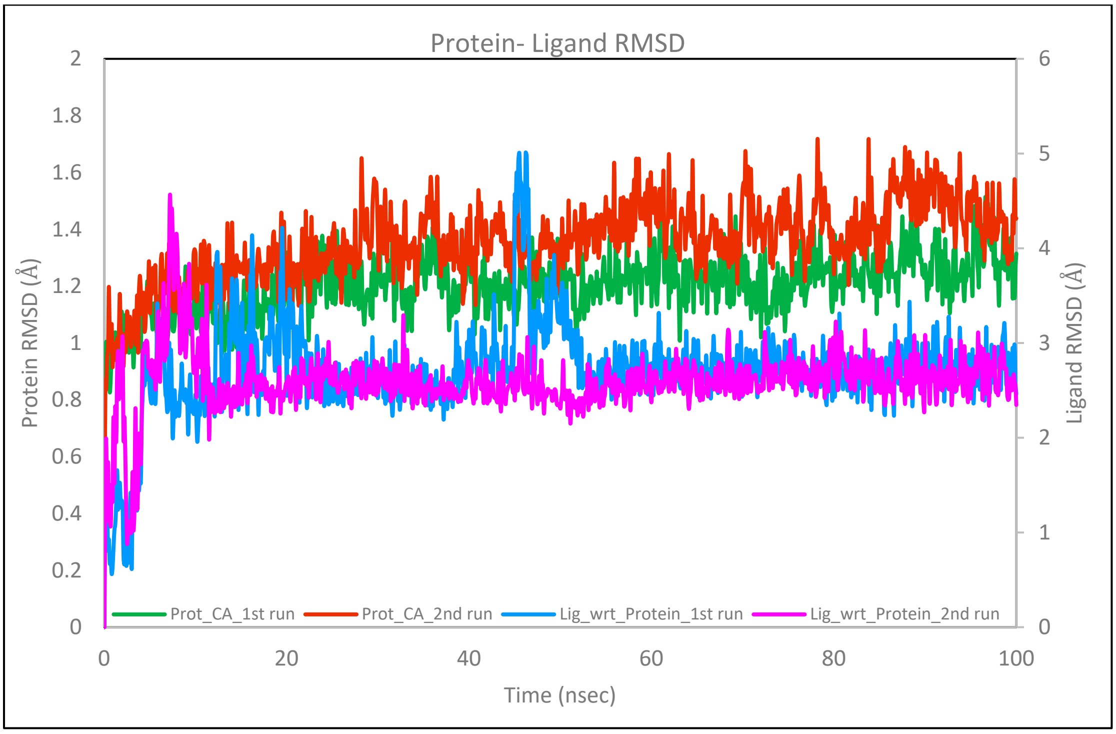

Figure S11. RMSD plot in duplicate for Quercetagetin-7-O-b-D-glucopyranoside (2) with pancreatic alpha-amylase (PDB-ID: 4GQR) during the MD simulation. Green and red represent the first and second run of protein, respectively. While blue and pink represent the first and second run of ligand

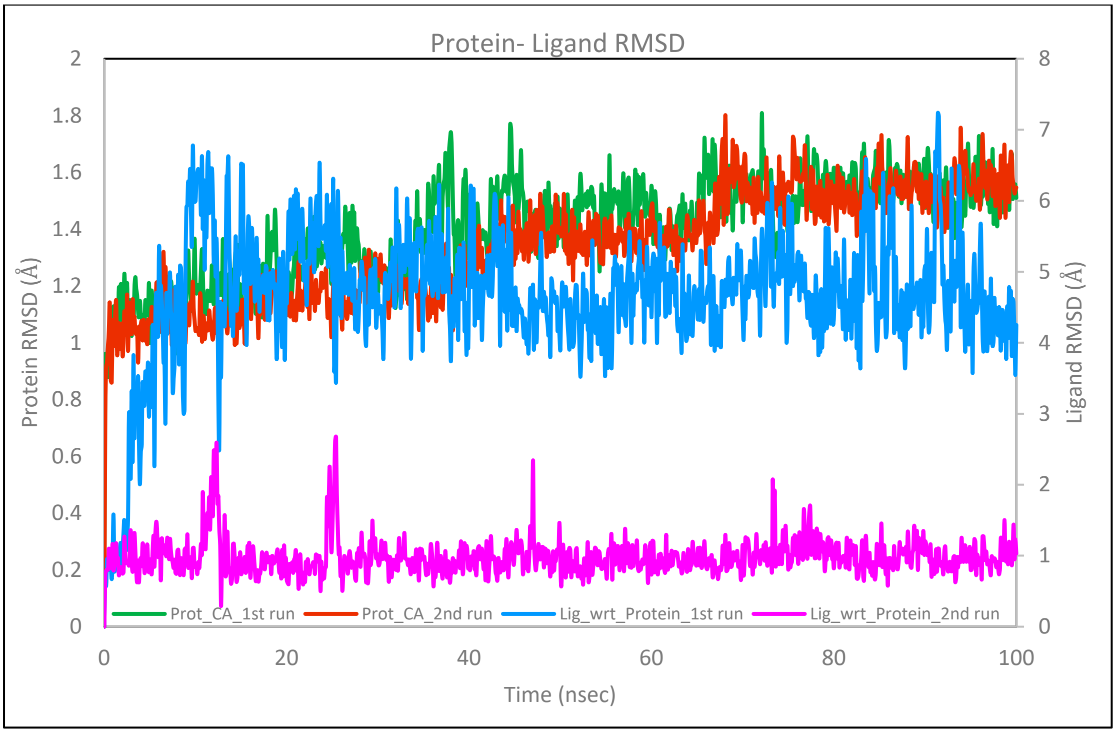

Figure S12. RMSD plot in duplicate for 3- Quercetagetin-6-O-b-D-glucopyranoside (3) with pancreatic alpha-amylase (PDB-ID: 4GQR) during the MD simulation Green and red represent the first and second run of protein, respectively. While blue and pink represent the first and second run of ligand

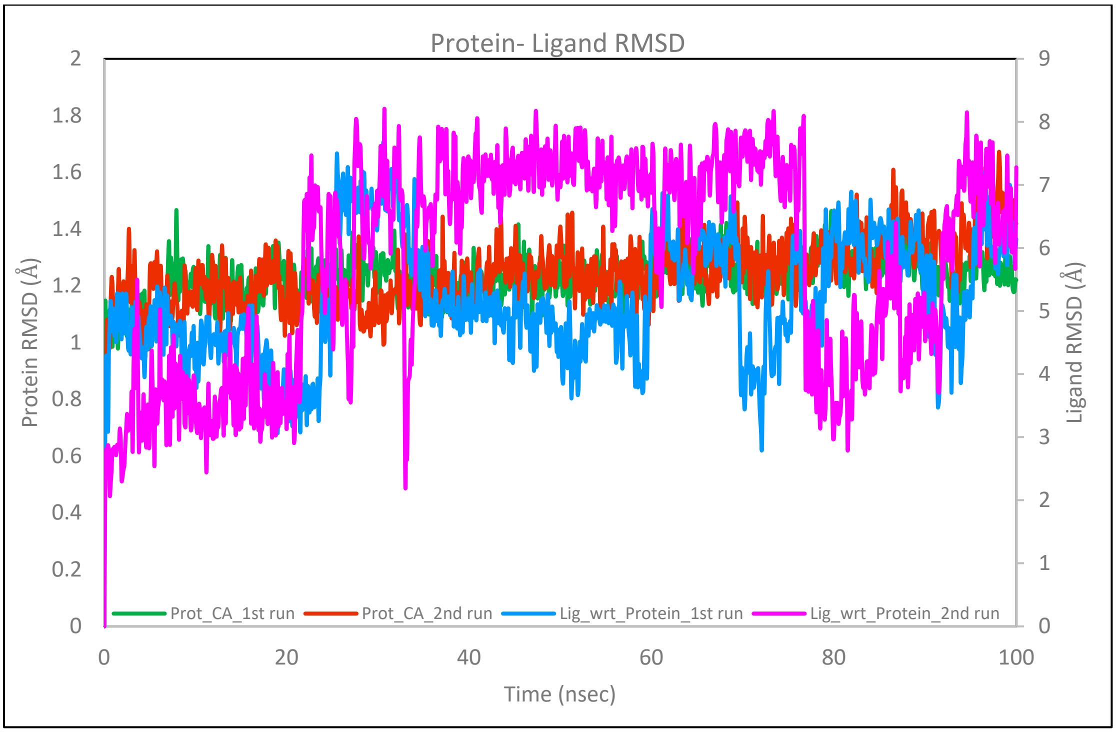

Figure S13. RMSD plot in duplicate for 4- MinuStructure574Minutaside A (4) with pancreatic alpha-amylase (PDB-ID: 4GQR) during the MD simulation Green and red represent the first and second run of protein, respectively. While blue and pink represent the first and second run of ligand

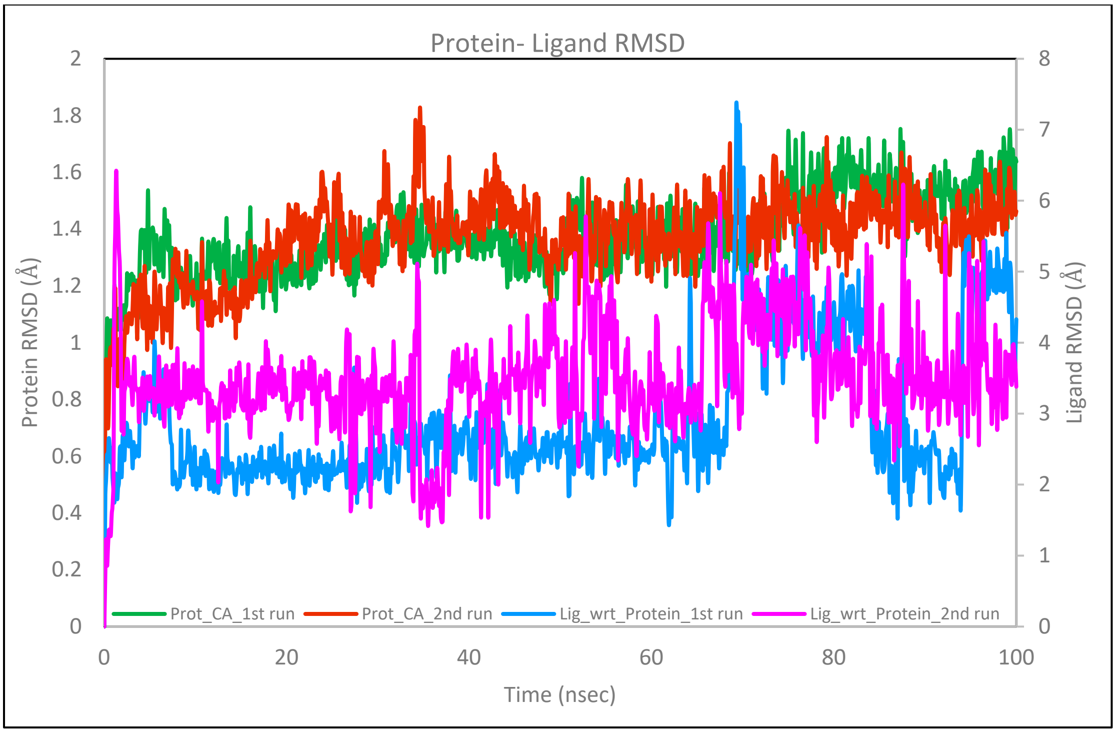

Figure S14. RMSD plot in duplicate for Patuletin-7-O-b-D-glucopyranoside (5) with pancreatic alpha-amylase (PDB-ID: 4GQR) during the MD simulation Green and red represent the first and second run of protein, respectively. While blue and pink represent the first and second run of ligand

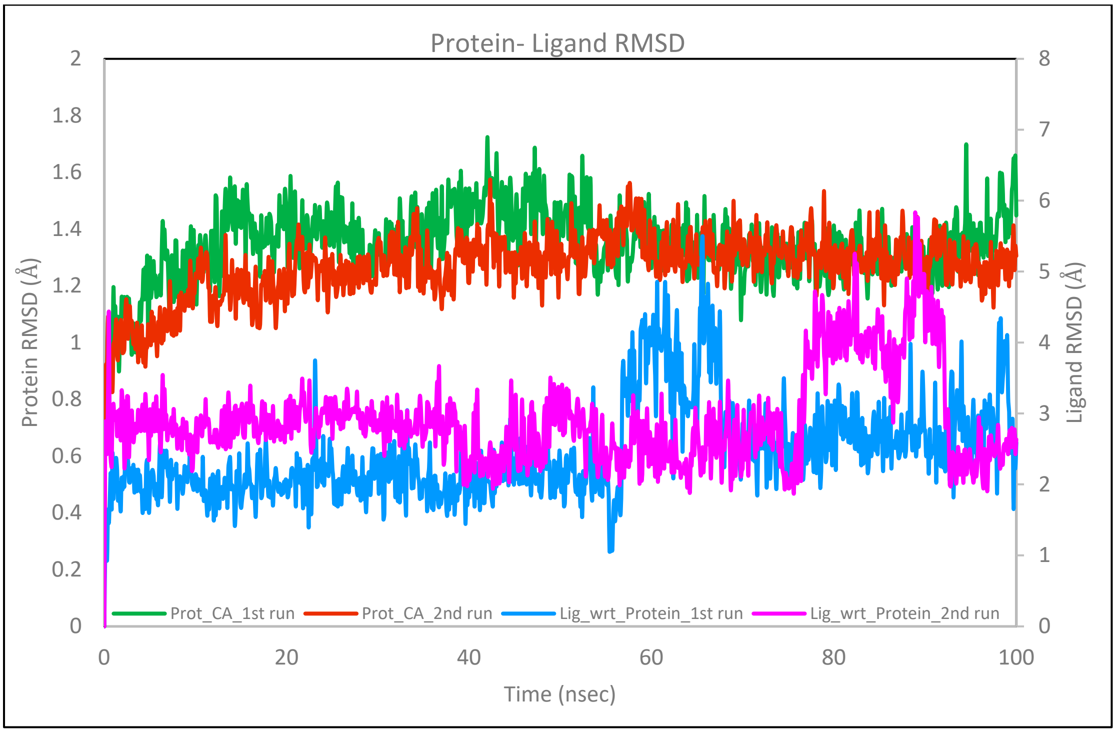

Figure S15. RMSD plot in duplicate for Quercetagetin-7-methoxy-6-O-b-D-glucopyranoside (6) with pancreatic alpha-amylase (PDB-ID: 4GQR) during the MD simulation Green and red represent the first and second run of protein, respectively. While blue and pink represent the first and second run of ligand

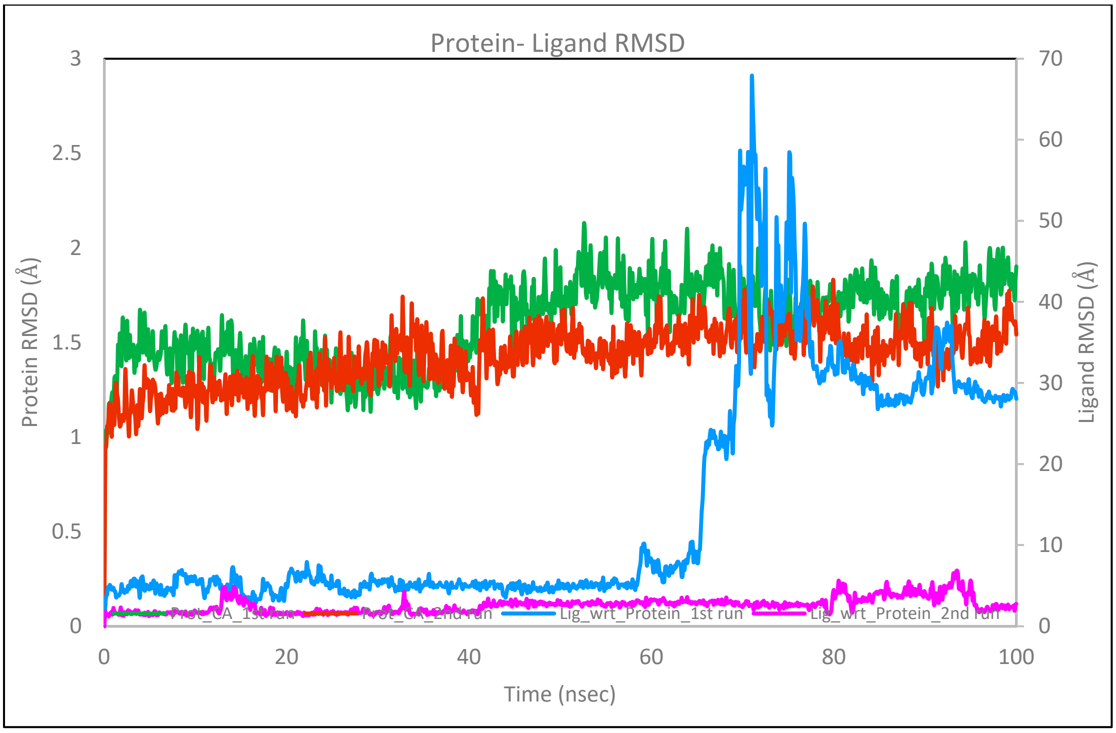

Figure S16. RMSD plot in duplicate for Acarbose with pancreatic alpha-amylase (PDB-ID: 4GQR) during the MD simulation Green and red represent the first and second run of protein, respectively. While blue and pink represent the first and second run of ligand

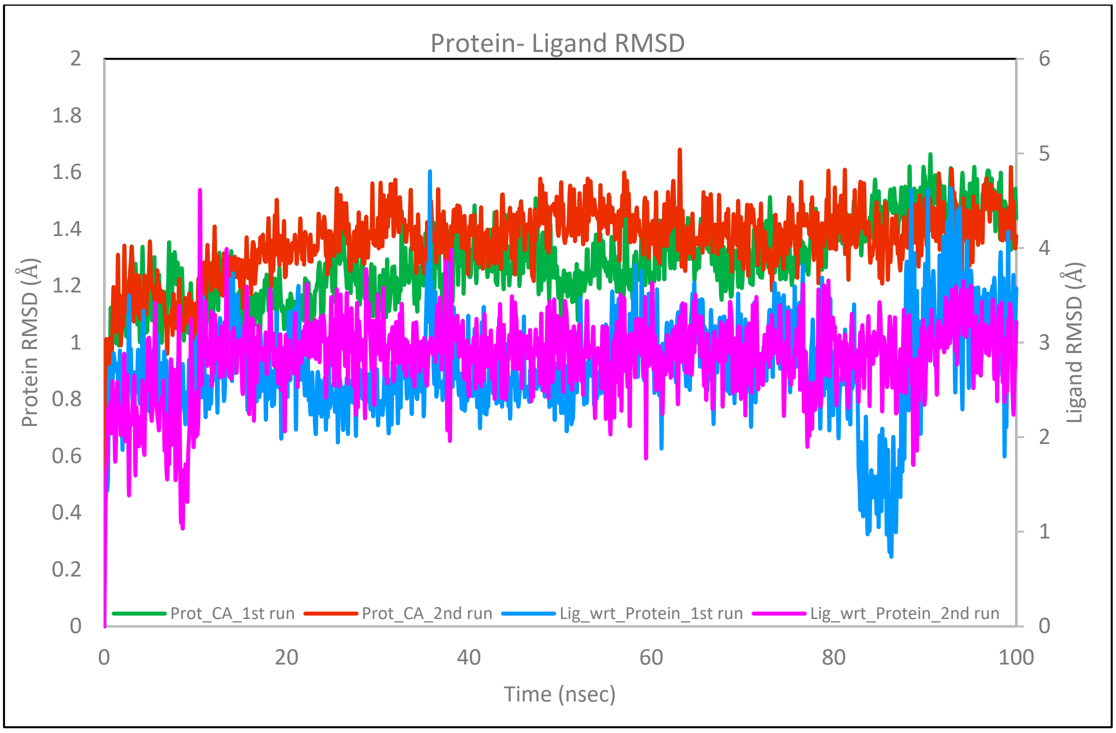

Figure S17. RMSD plot in duplicate for Myricetin with pancreatic alpha-amylase (PDB-ID: 4GQR) during the MD simulation Green and red represent the first and second run of protein, respectively. While blue and pink represent the first and second run of ligand
